# Ultrascalable Surface Structuring Strategy of Metal Additively Manufactured Materials for Enhanced Condensation

**DOI:** 10.1002/advs.202104454

**Published:** 2022-07-03

**Authors:** Jin Yao Ho, Kazi Fazle Rabbi, Siavash Khodakarami, Soumyadip Sett, Teck Neng Wong, Kai Choong Leong, William P King, Nenad Miljkovic

**Affiliations:** ^1^ Department of Mechanical Science and Engineering University of Illinois at Urbana‐Champaign Urbana IL 61801 USA; ^2^ Singapore Centre for 3D Printing School of Mechanical and Aerospace Engineering Nanyang Technological University 50 Nanyang Avenue Singapore 639798 Singapore; ^3^ Department of Electrical and Computer Engineering University of Illinois at Urbana‐Champaign Urbana IL 61801 USA; ^4^ Materials Research Laboratory University of Illinois at Urbana‐Champaign Urbana IL 61801 USA; ^5^ International Institute for Carbon Neutral Energy Research (WPI‐I2CNER) Kyushu University 744 Moto‐oka Nishi‐ku Fukuoka 819‐0395 Japan

**Keywords:** additive manufacturing, condensation, nanostructure, surface engineering, wettability

## Abstract

Metal additive manufacturing (AM) enables unparalleled design freedom for the development of optimized devices in a plethora of applications. The requirement for the use of nonconventional aluminum alloys such as AlSi10Mg has made the rational micro/nanostructuring of metal AM challenging. Here, the techniques are developed and the fundamental mechanisms governing the micro/nanostructuring of AlSi10Mg, the most common metal AM material, are investigated. A surface structuring technique is rationally devised to form previously unexplored two‐tier nanoscale architectures that enable remarkably low adhesion, excellent resilience to condensation flooding, and enhanced liquid–vapor phase transition. Using condensation as a demonstration framework, it is shown that the two‐tier nanostructures achieve 6× higher heat transfer coefficient when compared to the best filmwise condensation. The study demonstrates that AM‐enabled nanostructuring is optimal for confining droplets while reducing adhesion to facilitate droplet detachment. Extensive benchmarking with past reported data shows that the demonstrated heat transfer enhancement has not been achieved previously under high supersaturation conditions using conventional aluminum, further motivating the need for AM nanostructures. Finally, it has been demonstrated that the synergistic combination of wide AM design freedom and optimal AM nanostructuring method can provide an ultracompact condenser having excellent thermal performance and power density.

## Graphical abstract

Additive manufacturing enables previously unexplored two‐tier structured surface morphologies that exhibit unprecedented liquid repellency and phase change thermal transport efficiency not capable with conventional metal alloys. The synergistic combination of additive manufacturing macroscale design freedom with the developed nanostructuring methodology enables the multiscale codesign for optimization of device efficiency, power, and energy density.

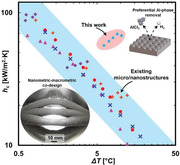

## 1 Introduction

Surface micro and nanostructuring plays a vital role in tuning surface wettability to govern interfacial transport mechanisms for mass, momentum and energy. When appropriately designed, the inclusion of surface structures can significantly enhance two‐phase heat and mass transfer performance, benefiting many industrial processes such as water harvesting,^[^ ^1^ ^]^ power generation,^[^ ^2^ ^]^ electrified transportation,^[^ ^3^ ^]^ electronics thermal management,^[^ ^4^ ^]^ refrigeration,^[^ ^5^ ^]^ and petrochemical operations.^[^ ^6^ ^]^ While the majority of past work has focused on generating dense nanostructures^[^ ^7^, ^8^, ^9^, ^10^, ^11^, ^12^, ^13^, ^14^ ^]^ to improve droplet repellency and mobility by preventing water droplets from penetrating the interstructure spacings, these strategies are not effective in harsh environments such as in pure vapor conditions with high supersaturations. Furthermore, even for favorable conditions where droplets form in nonwetting states, voids beneath suspended droplets act as an additional barrier for heat and mass transfer.^[^ ^15^ ^]^ Surface micro/nanostructures having mixed wettability^[^ ^16^, ^17^, ^18^, ^19^, ^20^ ^]^ allow for the spatial control of heterogeneous nucleation, preventing the transition to wetting states while also increasing nucleation site density. However, fabrication of mixed wettability interfaces requires techniques that are expensive and not easily scalable. Low‐cost and highly scalable surface structuring methods are essential for enabling implementation in large scale thermal systems, offering a viable approach to address technological challenges by enhancing heat and mass transport efficiency.

Despite recent progress in developing scalable surface nanostructuring techniques on conventional materials and alloys,^[^ ^9^, ^10^, ^21^, ^22^ ^]^ the recent proliferation of metal additive manufacturing (AM) as a viable manufacturing approach^[^ ^23^, ^24^, ^25^, ^26^ ^]^ has introduced additional materials‐related challenges. AM has received significant attention in recent years for producing functional parts in aerospace, electronics packaging, biomedical and construction industries^[^ ^23^, ^24^, ^25^, ^26^ ^]^ due to its design freedom and versatility. AM has the potential to fabricate and customize parts with highly complex shapes^[^ ^27^, ^28^ ^]^ that are not achievable by conventional manufacturing techniques. In particular, AM Al‐alloy (AlSi10Mg), one of the most popular metal AM materials, has good thermal as well as mechanical properties and is suitable for powder bed fusion AM processes. Resulting from these properties, this alloy is one of the most widely used metal AM material in a plethora of applications such as aircraft components, compact heat exchangers, and energy storage systems. Despite more than a decade of intensive research, scalable nanostructuring strategies for AM alloy surfaces with robust superhydrophobicity have yet to be demonstrated. Given the growing relevance of metal AM manufacturing processes, an urgent need exists to develop rational nanostructuring strategies. A successful synergistic combination of surface structuring with the complex and co‐optimized design methodology of AM will not only benefit the energy sector by enabling enhanced two‐phase heat and mass transfer on nanostructured AM components, but will also have profound impact on the aerospace, transportation, biomedical, and construction industries, just to name a few, where surface superhydrophobicity, superhydrophilicity and oleophobicity are important.

In this work, we take advantage of the intrinsic material properties of AlSi10Mg alloy to develop preferential etching and chemical oxidation processes for ultrascalable production of intrinsic 2D cellular nanostructures unique to metal AM surfaces. The resulting 3D two‐tier nanostructures consists of a first‐tier cellular network which allows droplet formation within cell cavities while compartmentalizing them to prevent lateral spreading and a second‐tier intersected platelet structures of smaller feature sizes which serve to reinforce droplet confinement and reduce adhesion. Using condensation as a demonstration framework, we experimentally show that the AM Al‐alloy with two‐tier nanostructured surface is capable of sustaining ultrastable droplet self‐repellency via coalescence‐induced droplet jumping even at high supersaturation (*S* ≈ 1.8), resulting in a 6X and 2X higher condensation heat transfer coefficients (HTC) as compared to filmwise and dropwise condensation, respectively, on nanostructured conventionally manufactured aluminum alloy surfaces. To demonstrate the clear advantage of our AM structuring technique, we further fabricated a functional multitube nanostructured compact AM heat exchanger which showed stable droplet jumping condensation at high supersaturations along with jumping droplet induced inter‐tube droplet sweeping. These results exhibit the clear and remarkable potential of integrating nanostructuring with the AM design process for the creation of codesigned and truly optimized devices for a plethora of energy applications such as heat pumps,^[^ ^3^ ^]^ surface condensers^[^ ^29^ ^]^ and electronics cooling systems.^[^ ^30^ ^]^

## 2 Results

**Figure 1:**
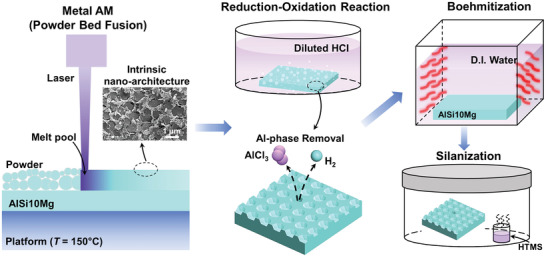
AM fabrication and surface structuring process. Schematic showing metal AM powder bed fusion fabrication resulting in intrinsic subgrain nanoarchitecture consisting of Al‐rich cell core and Si‐rich cell wall, preferential removal of Al‐phase by HCl etchant resulting in 3D nanocellular structures, boehmitization process to develop second‐tier nanostructure and silanization process to produce superhydrophobic AM surfaces.

Selective laser melting (SLM) is a powder bed fusion AM technique which utilizes a laser source to selectively melt the base metallic powder located on a low temperature platform (**Figure** **1**). As the powder melts, ultrathin molten pools (≈50 µm) are formed which then quickly cool and solidify, forming a dense solid layer. Stemming from the novel AM alloying materials and rapid solidification process, unique nanoscale subgrain structures, previously unachievable in conventionally processed metals, are produced due to the epitaxial growth of crystals. For example, AlSi10Mg AM material leads to the formation of a novel nano‐sized cellular network with Si‐rich aluminum–silicon (Al–Si) cell walls, and Al‐rich Al–Si cell cores^[^ ^36^, ^37^, ^38^, ^39^ ^]^ (see Figure 1 and Figure S7 in the Supporting Information for illustration of the nanocell network). The presence of cellular subgrain structures on AM samples has also been verified through the use of the well‐established Keller's reagent (see Section S3 of the Supporting Information for details). Therefore, existing AM surface structuring challenges can be overcome if a 3D structuring technique can be rationally devised by taking advantage of the intrinsic 2D nanoarchitecture of AM manufactured parts.

Here, we use AlSi10Mg as a rational material selection because of three reasons: 1) micro and nanostructuring of the Al phase can be easily achieved using conventional methods due to the high chemical reactivity of pure Al when compared to other widely used metals, 2) the presence of Si as an alloying element represents a less reactive secondary phase, resulting in the potential to fabricate cellular‐like nanostructures, and 3) the ubiquitous use of Al‐alloys in many industrial applications due to the their excellent thermophysical properties and relatively low density. Here, we use the SLM280HL AM facility to fabricate samples with a laser power of 200 W, scanning speed of 1.3 m s^−1^ and a hatch spacing of 80 µm, selected based on previous fabrication of bulk components with good structural integrity^[^ ^27^, ^28^, ^31^, ^32^, ^33^ ^]^ (see Section S2 of the Supporting Information for the detailed AM fabrication process). Flat AM samples having 1 in. × 1 in. area were fabricated for the characterization of their surface morphology and wetting performance.

To generate 3D structures for AM, we adopt a top‐down structuring approach by performing chemical etching on the AM samples (Figure 1). Hydrochloric acid (HCl) solution is rationally selected as the etchant due to its high reactivity with Al. We hypothesize that HCl etching will preferentially remove the Al‐rich phase of the intrinsic subgrain structures but not the Si‐rich cell walls, thereby generating a previously unexplored type of 3D cellular architecture. To test out our hypothesis and obtain further understanding of the etching mechanisms, we immersed as‐fabricated AM samples in a 2.0 M HCl solution and varied the etched time between 0 and 6 min. Prior to etching, all samples were thoroughly cleaned by immersing them successively in acetone, ethanol and isopropyl alcohol for 10 min in an ultrasonic bath followed by rinsing with deionized (DI) water. After etching, each sample was functionalized by chemical vapor deposition of heptadecafluorodecyl‐trimethoxy‐silane (HTMS) at atmospheric pressure using 5% v/v of HTMS‐toluene in a sealed container maintained at 90 °C inside an atmospheric pressure furnace for 3 hours.

**Figure 2:**
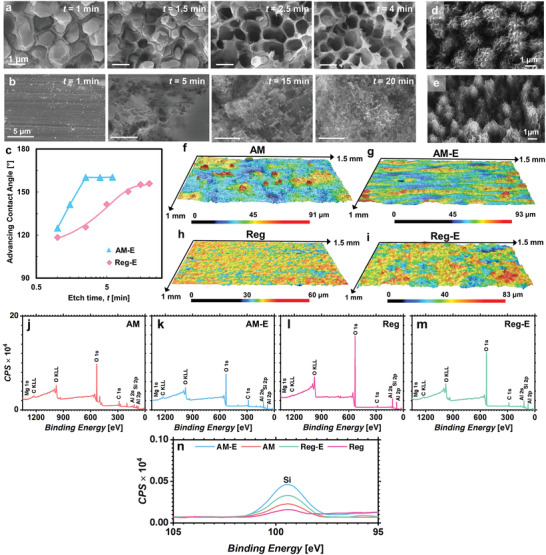
Characterization of the structured surfaces. Top view SEM images of micro/nanostructures on: a) additively manufactured etched surface (AM‐E) at different etch time (*t*) and b) conventional etched surface (Reg‐E) at different etch time (*t*). c) Advancing contact angles of AM‐E and Reg‐E at different etch time (*t*). SEM images of two‐tier nanostructures on additive manufactured etched and boehmite surface (AM‐EB) d) top view and e) side view. Confocal microscopy images illustrating the roughness profile of AM surface f) before and g) after etching for 2.5 min with 2 m HCl, and conventional Al‐6061 surface h) before and i) after etching for 15 min. The root‐mean‐square roughness (*R*_rms_) of Al‐6061 before and after etching is ±3.3 µm and ±10.7 µm, respectively whereas *R*_rms_ of AM before and after etching is ±9.2 and ±7.2 µm, respectively. XPS elemental survey scan spectra showing the material composition for the j) unaltered additively manufactured surface (AM), k) AM etched surface (AM‐E), l) conventional Al surface (Reg), and m) conventional etched Al surface (Reg‐E). n) High resolution XPS scan of the Si 2p spectra showing the silicon content comparison on the four surfaces.

**Figure** **2**a shows scanning electron microscopy (SEM) images of the AM samples (AM‐E) after each etching interval. Etching for 1 min only reveals the shallow cell structures. For 1.5 min etch durations, the Al‐rich core begins to react with the etchant and variation in the pore depth are observed. For 1.5 min etch times, some cells show completely removed Al‐rich cores while some cell cores remain partially etched. By further increasing the etching time, cellular structures with deeper cell core and slender Si‐rich walls become visible. These SEM results confirm our hypothesis of the etching mechanism between HCl and Al leading to the preferential removal of the Al‐rich cell core and formation of unique three‐dimensional nano‐cells. Using SEM and focused ion beam (FIB) milling (see Figures S3 to S5 in the Supporting Information), each cell was estimated to have a pore size of ≈1 µm, depth of ≈1.5 µm and is bounded by cell walls that are 50–100 nm thick. To further demonstrate that an optimized etch time was achieved, we performed goniometric measurements to quantify wetting characteristics of each HTMS‐functionalized sample (see Section S3 in the Supporting Information). Figure 2c shows the variation of the apparent advancing contact angle using deionized water droplets as a function of etch time. The advancing contact angle initially increases with etch time (*t*) but stabilizes at ≈160° after *t* ≥ 2.5 min.

To provide clear evidence that the observed unique nanostructures can only be generated on AM surfaces, we carried identical etching procedures on the most commonly used conventional Al alloy, Al‐6061 by ranging the etch time between 0 and 20 min. Figure 2b shows that conventional Al‐6061 samples (Reg‐E) consist of highly irregular microscale cavities^[^ ^34^ ^]^ that are distinctly different from the AM‐E samples. Further goniometric characterization on the etched and functionalized Al‐6061 samples (Figure 2b) reveals that the optimized etch time of Reg‐E is 15 min where the advancing contact angle stabilizes at ≈155°. To characterize the macroscale roughness of the samples, we performed high‐resolution confocal microscopy analysis (see Section S3 in the Supporting Information). As shown in Figures 2f–i, our results show that while the macroscale rms roughness of conventional Al‐6061 (Reg) substantially increases after etching, the rms roughness of AM samples reduces. This is primarily because the etching process not only generates cellular nanostructures, but, in the process, removes un‐melted AM powder from the top surface.

To verify the observed etching mechanism, we measured eight spatially random locations on the etched AM cell walls using energy‐dispersive X‐ray spectroscopy (EDS, see Section S3 in the Supporting Information). The average concentration of each element is shown in **Table** **1**. The Si concentration (14.16 wt%) on the etched AM surface is higher than the unetched AM surface (8.93 wt%). To further verify the presence of Si‐rich cell walls, we performed X‐ray photoelectron spectroscopy (XPS) on the etched and unetched surfaces (see Section S3 of the Supporting Information for method). As XPS analysis allows for the measurement of the surface material composition in the nanometric depth range, it can reveal the composition of the cell walls with a higher accuracy. Figure 2j–n shows the XPS elemental survey scan spectra between 0 and 1350 eV binding energy showing the surface elemental contents (Mg, C, Al, O, Si, Al) at their corresponding binding energies for the unaltered additively manufactured surface (AM), AM etched (AM‐E) surface, conventional Al (Reg) surface, and conventional etched Al (Reg‐E) surface, respectively. Peaks at 284.8 and 532.5 eV are from adventitious carbon and native oxide, respectively, due to surface exposure to the atmosphere. Comparative atomic percentage analysis between aluminum (Al), silicon (Si), and magnesium (Mg) from the XPS elemental survey scans is presented in **Table** **2**. Table 2 shows that the atomic percentage breakdown for the AM surface agrees well with the AlSi10Mg elemental composition with the percentage of Si increasing on both the etched AM‐E as well as the Reg‐E surfaces. Furthermore, Table 2 illustrates the negligible amount of Si on the conventional Al‐6061 surface. The increased amount of Si on the etched Reg‐E surface is smaller compared to the AM‐E surface. Figure 2n shows the high resolution XPS scan for the Si 2p spectra illustrating the comparative presence of Si (Si 2p at 99.4 eV) content on the four surfaces. Figure 2n shows that a higher amount of Si exists on both etched AM and etched conventional Al surfaces when compared to their un‐etched counterparts. It should be noted that Mg reacts with Si to form magnesium silicide (Mg_2_Si) during the AM fabrication process which mainly forms at the cell boundaries.^[^ ^36^, ^37^, ^38^ ^]^ As Mg_2_Si reacts with HCl, a certain amount of Mg_2_Si is removed during the chemical etching process. However, since the concentration of Mg is small (≈1 wt%), its influence on the nanostructure morphology was observed to be insignificant.

**Table 1:** Elemental composition of the AM and conventional Al‐alloy surfaces before and after etching obtained from EDS analysis

	AM AlSI10Mg				Conventional Al 6061			
Elements	Al	Si	O	Mg	Al	Si	O	Mg
Unetched [conc. wt%]	85.08	8.93	4.98	1.01	96.85	0.7	0.8	0.9
Etched (conc. wt%)	79.68	14.16	5.24	0.93	93.48	0.94	0.21	1.96

**Table 2:** Elemental composition of the AM and conventional Al‐alloy surfaces before and after etching obtained from XPS analysis

	AM AlSI10Mg			Conventional Al 6061		
Elements	Al 2p	Si 2p	Mg 1s	Al 2p	Si 2p	Mg 1s
Unetched [at%]	88.89	11.11	≪1	≈100	≪1	≪1
Etched [at%]	67.35	32.22	≪1	93.31	6.69	≪1

The presence of substantial amounts of Al in the Si‐rich wall of the AM‐E sample after etching (see Tables 1 and 2) points out a further advantage of AM processing by enabling the development of a second‐tier structure on top of the existing cellular network using bottom‐up approaches such as oxidation.^[^ ^35^ ^]^ To demonstrate this advantage, we adopt the boehmitization structuring process commonly used for aluminum alloys to construct the second tier nanoarchitecture on AM‐E. Boehmitization is achieved by immersing AM‐E into a hot DI water bath for 30 min at the water temperature of 95 ± 2 °C. The hot water causes a self‐limiting reaction at the Al‐phase surfaces where the surface reaction will self‐saturate and stabilize when the surface reaction site depletes with increasing exposure time. This reaction resulted in the formation of a thin layer of boehmite (AlO(OH)) having ≈300 nm length scale, forming second‐tier nanostructures, termed AM‐EB herein. Note that the height of the boehmite structure is determined from the SEM images of the cross section of the surface structures obtained by FIB (see Figure S4 of the Supporting Information). Figure 2d,e shows the top and side views of the AM‐EB structures. Despite the high Si concentration, the presence of the Al phase enables the formation of a dense boehmite layer on the cell walls and within the cellular structure, which further reduces the cellular pore size (see Figure S1d,e in the Supporting Information). Using micro‐goniometry, the advancing contact angle of deionized water droplets on the AM‐EB sample approached 163°. This contact angle is slightly higher than on the AM‐E sample due to the reduced solid‐liquid interface fraction enabled by the smaller boehmite structures.

To verify the effectiveness of the superhydrophobic AM‐E and AM‐EB surfaces, we further fabricated a boehmitized single‐tier superhydrophobic AM tube, termed AM‐B. Furthermore, to compare the performance of the AM surfaces with conventional Al‐alloy tubes, three superhydrophobic Al‐6061 surfaces having identical dimensions were prepared with one boehmitized, one chemically etched, and one treated with the two‐step process, termed Reg‐B, Reg‐E, and Reg‐EB, respectively (see Section S2 and Figures S3 and S5 of the Supporting Information for details regarding the fabrication process and SEM side and top views of the micro/nanostructures). To investigate samples representative of typical heat exchanger geometries, AM and conventional Al‐6061 tubes with outer diameters (OD) of 12.7 mm, inner diameters (ID) of 10.7 mm and lengths of 266.7 mm were further fabricated using the same surface treatment processes discussed above (see Section S2 and Figure S1 of the Supporting Information for fabrication details).

The wetting properties of the surfaces were determined by goniometric characterization. The apparent advancing (*θ*_a_) and receding (*θ*_r_) contact angles were determined using a microgoniometer (see Supporting Information S3) with 100 nL water droplets dispensed on the treated AM and conventional Al samples. Each contact angle was determined from at least three spatially random measurements, with an average of 10 sampling points at each location. Note that *θ*_r_ is obtained during droplet evaporation. The measured contact angles are summarized in **Table** **3** along with a summary of treatment time of the samples. All samples exhibited excellent superhydrophobicity, with *θ*_a_ and *θ*_r_ greater than 150°, and low contact angle hysteresis (Δ*θ* = *θ*_a_ − *θ*_r_).

**Table 3:** Measured apparent advancing (*θ*_a_) and receding (*θ*_r_) contact angles

Sample	Etch time [min]	Boehmitization time [min]	Silanization time [h]	*θ*_a_ [°]	*θ*_r_ [°]	Δ*θ* [°]
AM‐EB	2.5	30	3	163.0	161.3	1.6
AM‐E	2.5	0	3	160.7	159.2	1.5
AM‐B	0	30	3	154.9	153.6	1.3
Reg‐EB	15	30	3	161.4	160.0	1.4
Reg‐E	15	0	3	155.2	153.9	1.3
Reg‐B	0	30	3	163.3	162.0	1.3

To demonstrate the potential of our two‐tier micro and nanostructuring method of the AM Al‐alloy, we conducted steam condensation experiments. To quantify the two‐phase heat transfer performance, we determined the overall heat transfer coefficient (HTC) during condensation. To conduct the experiment, each sample tube was housed in a controlled condensation chamber (Figure S8 in the Supporting Information). Prior to the experiments, ambient air in the chamber was evacuated to a chamber pressure of *P* < 6 ± 2 Pa and DI water in the vapor generator was vigorously boiled under ambient pressure. These processes were carried out to eliminate noncondensable gases which cause additional diffusional resistances and affect the heat transfer measurement.^[^ ^40^ ^]^ During the condensation experiments, the chamber pressure and temperature were continuously monitored to ensure that the steam was at saturation conditions. The surface temperature of the tube sample was separately controlled by a standalone cooling water loop with the tube inlet water temperature maintained at 7 ^○^C < *T*_in_ < 8 ^○^C and water flow rate of 20.5 ± 0.2 to 30.6 ± 0.2 L min^−1^. At these flow rates, a fully developed turbulent internal flow with the Reynolds number (Re_D_) up to 44 000 was attained. The condensation heat flux was determined from measurement of the inlet and outlet coolant water temperatures, as well as the water flow rate by two Class A resistance temperature detectors (RTD) and an electromagnetic flow meter, respectively. To investigate the condensation performance of the samples for a wider range of applications and to examine the ability of the structures to sustain stable jumping droplet condensation, our experiments were conducted over a large range of vapor pressures (3.2 kPa < *P*_v_ < 7.4 kPa). At these vapor pressures, the corresponding vapor saturation temperature (*T*_sat_) ranged from 25 to 40 °C. The supersaturation *S*, defined as the ratio of vapor pressure to saturation pressure corresponding to the sample surface temperature (*S* = *P*_v_/*P*_w_), ranged from 1.4 to 2.0. For additional details of the experimental facility and test procedures, see Section S4 of the Supporting Information.

**Figure 3:**
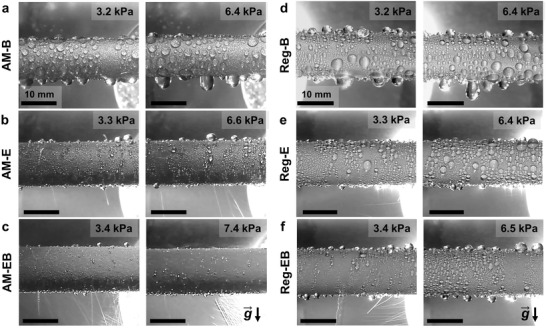
Optical images of steam condensation on tube samples. Images of steam condensing on a) AM‐B at *P*_v_ = 3.2 kPa (*S* = 1.6), and 6.4 kPa (*S* = 1.9), showing surface flooding and formation of highly pinned Wenzel droplets; b) AM‐E at *P*_v_ = 3.3 kPa (*S* = 1.4), and 6.6 kPa (*S* = 1.7), showing combination of droplet jumping and droplets rolling off the surface; c) AM‐EB at *P*_v_ = 3.4 kPa (*S* = 1.4), and 7.4 kPa (*S* = 1.8), showing highly mobile droplets leading to intense droplet jumping. Images of water condensing on d) Reg‐B at *P*_v_ = 3.2 kPa (*S* = 1.6), and 6.4 kPa (*S* = 1.9), showing surface flooding and formation of highly pinned Wenzel droplets; e) Reg‐E at *P*_v_ = 3.3 kPa (*S* = 1.5), and 6.6 kPa (*S* = 1.7), f) Reg‐EB at *P*_v_ = 3.4 kPa (*S* = 1.4), and 7.4 kPa (*S* = 1.8), showing mixture of droplet jumping and droplets rolling off the surface. See Video S2 in the Supporting Information for real time videos of steam condensing on all tube samples for vapor pressures ranging from 3.2 to 7.4 kPa.

**Figure** **3**a–c shows photographs of steam condensation on the AM tubes at various vapor pressures taken using a digital single‐lens reflex (DSLR) camera (Canon EOS T3i). In our experiments, the inlet cooling water temperature was kept constant, and hence the increase in vapor pressure corresponds to an increase in supersaturation. At each vapor pressure setting, images were taken after the system had stabilized for more than 10 min. Due to the presence of functionalized nanostructures and surface superhydrophobicity, discrete water droplets formed on the tubes. However, distinctly different droplet distribution sizes were observed on the various AM tubes. Droplets on the AM‐B surface (Figure 3a) were significantly larger and grew to sizes approaching the capillary length before removal by gravity. Droplet sizes approaching the capillary length are indicative of formation of highly pinned Wenzel droplet which wet the nanostructure cavities, resulting in poor mobility. High resolution SEM images of the AM‐B sample revealed the presence of dense boehmite nanostructures on the surface. Such structures are analogous to intersected platelets with average line widths of 25–30 nm and heights of 200–400 nm (see Figures S3e and S5e in the Supporting Information) and are found to promote droplet jumping at low supersaturations of ≈1.06. However, condensation at high supersaturations on the boehmite nanostructures cannot ensure confinement of the droplet base, leading to flooding. Furthermore, the rapid melting and solidification of the metallic powder during SLM results in the formation of unmelted granular particles on the sample surface. SEM images revealed that boehmite does not form on these particles (Figure S6 in the Supporting Information) because of their low Al content, making these regions preferential droplet pinning sites (Figure 3a and Figure S11c, Supporting Information).

In contrast, the AM‐E tube exhibited significantly higher droplet mobility. A mixture of droplet shedding mechanisms coexisted, where droplets spontaneously jumped and rolled off the surface. Jumping‐droplet‐condensation on the AM‐E tube is evident from the visible light streaks of the droplet jumping trajectories in Figure 3b. Droplet jumping was observed for the entire range of tested vapor pressures (3.3 kPa < *P*_v_ < 6.6 kPa), corresponding to 1.4 < *S* < 1.7. The size of pinned nonjumping droplets on the AM‐E surface was significantly smaller and exhibited higher apparent advancing contact angle when compared to droplets on the AM‐B surface.

The excellent jumping and antiflooding performance of the AM‐E sample is due to its unique cellular‐like nanostructures (Figures 2a and Figure S3b, Supporting Information). When a nanodroplet nucleates within the cellular cavity, a high local energy barrier imposed by the cell walls limits lateral growth^[^ ^41^ ^]^ and forces the droplet to grow in the upward direction resulting in the partially wetting state where a Cassie droplet sits atop the nanostructures and a confined liquid‐filled region exists beneath the droplet within the cellular cavity. It should be noted that the droplet nucleation size (*r*_min_) during condensation reduces with increasing supersaturation, where *r*_min_ = 2*T*_sat_ *σ*/*h*_fg_ *ρ*_l_Δ*T*.^[^ ^9^, ^10^ ^]^ In this study, the condensation experiments were performed for *P*_sat_ ranging from 3.2 to 7.4 kPa and Δ*T* ranging from 5 to 12 °C which correspond to *r*_min_ between 1.4 and 3.5 nm. Since *r*_min_ is much smaller than the nanostructure cavity size and the area of the cavity side walls are much larger than its top solid area, the probability of droplets nucleating within cavities is significantly higher when compared to the probability of droplets nucleating on top. However, regardless of the droplet nucleation size, as long as the droplet nucleates within the cellular cavity, its lateral growth will be constrained by the cell walls, which explains the stable droplet jumping achieved by the cellular structure even at high supersaturations.

Figure 3c shows images of steam condensation on the AM‐EB tube. For the entire range of tested vapor pressures, stable droplet‐jumping condensation was observed. Compared to the other surfaces shown in Figure 3, it is evident that the AM‐EB tube exhibits the best condensation characteristics with intense droplet jumping and a negligible amount of pinned liquid droplet on its surface for the entire range of tested vapor pressures. As depicted in Figure S3a,e the length scale of the boehmite is approximately one order of magnitude smaller than the cellular structure length scale. The combination of a dense secondary structure (boehmite) on the cell walls while still preserving the overall shape of the cellular structure, results in a structure topology that is advantageous for enhancing droplet jumping and preventing surface flooding. With a second tier boehmite phase forming on the cellular walls, the effective height of the structures further increases, providing an additional energy barrier to impede lateral growth (Figure S9a, Supporting Information). In addition, the two‐tier (cellular‐boehmite) structures have reduced droplet‐surface adhesion compared to the single tier cellular structures, resulting in more frequent droplet jumping. Considering two adjacent partially wetted droplets immediately prior to coalescence (Figure S10, Supporting Information), the work of adhesion is $W_{a}\;=\;2\sigma_{\mathrm{lv}}\left\{\varphi A_{b}\left(1+\cos\theta_{r}^{\mathrm{app}}\right)+2A_{p}\right\}$, where the first term in the brackets corresponds to the adhesion in the nonwetted region of the droplet–surface interface and the second term is associated with the adhesion of the wetted region^[^ ^8^, ^42^, ^43^ ^]^ (see Supporting Information S5). The term *A*_b_ denotes the droplet basal area, *ϕ* is the nanostructure solid fraction and *A*_p_ is the projected area of water‐filled cavity in contact with the droplet base. Hence, *ϕA*_b_ represents the contact area between the droplet base and the top surface of the nanostructure. For the AM‐E materials, *ϕ* = *ϕ*_AM−E_, which is the ratio of *a*_1_ to *c* in Figure S9b (Supporting Information), whereas for the AM‐EB materials, due to the secondary boehmite layer forming atop the cellular structures, the droplet‐nanostructure contact area is reduced, giving *ϕ* ≈ *ϕ*_AM−E_ · *ϕ*_AM−B_ (or ratio of *a*_2_ to *c* as shown in Figure S9b (Supporting Information). Hence, the AM‐EB sample has a lower adhesion associated with the nonwetted region. Furthermore, in the wetted region, the presence of boehmite on the inner cell walls prevents condensate from completely filling the cellular cavities (Figure S9b, Supporting Information). This reduces the water‐filled cavity area in contact with the droplet base (*A*_p_) and lowers the droplet adhesion to AM‐EB in the wetted region as compared to AM‐E.

**Figure 4:**
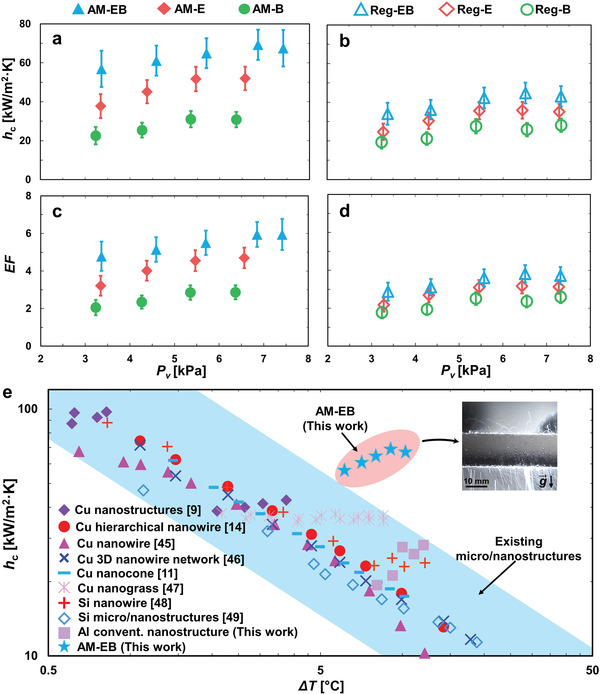
Measured condensation performance. Experimental steady‐state condensation heat transfer coefficient (*h*_c_) as a function of saturated vapor pressure (*P*_v_) for the a) AM‐EB, AM‐E and AM‐B, and b) Reg‐EB, Reg‐E, and Reg‐B tubes. All surfaces exhibited dropwise condensation with AM‐EB exhibiting the highest *h*_c_ due to jumping‐droplet dominated enhanced condensation for the entire range of *P*_v_. Condensation enhancement factor (EF) of c) AM‐EB, AM‐E and AM‐B, and d) Reg‐EM‐ Reg‐E and Reg‐B surfaces. The values of *EF* represent the *h*_c_ ratio of an enhanced tube to a plain tube exhibiting filmwise condensation at the same supersaturation (*S*). The *h*_c_ of filmwise condensation was determined from the classical Nusselt theory. The highest *EF* value of 5.95 was achieved by AM‐EB. The measurement uncertainties of *h*_c_ and *EF* range from 11.2% to 19.8%. e) Comparison of the jumping droplet condensation heat transfer coefficient (*h*_c_) of our AM‐EB surface with previously reported superhydrophobic surfaces with respect to subcooling (Δ*T*).

**Figure** 4a,b shows the average condensation heat transfer coefficient (*h*_c_) of the AM and conventional Al‐alloy tubes obtained from experimental measurements and plotted as a function of vapor pressure (*P*_v_). For a detailed description of data reduction with uncertainty analysis, see Section S4 of Supporting Information. A comparison of *h*_c_ in Figure 4a shows that AM‐EB has the best condensation performance amongst all the AM surfaces with *h*_c_ ranging from 56.9 to 69.3 ± 9.4 kW (m^−2^ K^−1^). The excellent condensation performance of the AM‐EB surface was mainly due to the small droplet departure size and high jumping frequency which allows continuous regeneration of nucleation sites further promoting microdroplet growth. In case of AM‐EB surface, the average droplet departure size is smaller, and presence of pinned droplets on the surface are negligible. As a result, for AM‐EB surface the droplet thermal resistances are significantly smaller compared to AM‐E and AM‐B. Furthermore, as the droplet jumping is consistent and stable throughout the entire range of vapor pressures (Figure 3c), no degradation in *h*_c_ was observed from the experimental results. In fact, a slight increase in *h*_c_ with increasing *P*_v_ was observed. The increased *h*_c_ is due to the increased nucleation density at elevated supersaturations, as well as the change in the interfacial mass transfer resistance which is sensitive to vapor pressure and becomes less dominant as vapor pressure increases.^[^ ^9^ ^]^

To compare the condensation performance of the AM tubes, we characterized the condensation heat transfer coefficient (*h*_c_) of the superhydrophobic tubes made from conventional Al‐6061 (Reg‐EB, Reg‐E, and Reg‐B). The measured *h*_c_ values of the conventional Al tubes are shown in Figure 4b (detailed images of condensation shown in Figure S11 in the Supporting Information S6). For Reg‐EB, droplet removal by coalescence‐induced jumping and droplet shedding were observed at low vapor pressures. However, as *P*_v_ increased above 4.4 kPa (*S* > 1.5), no jumping was observed, with nonwetting droplets transitioning to the pinned Wenzel state. Similarly, for Reg‐E and Reg‐B, droplet jumping was not observed for the range of tested vapor pressures (3.2 kPa < *P*_v_ < 7.2 kPa, 1.5 < *S* < 2.0) with highly pinned Wenzel droplets populating the tube surfaces. Accordingly, due to poorer droplet mobility on the conventional Al surfaces, their corresponding *h*_c_ values were lower when compared to the AM surfaces that underwent identical treatment processes. For both single‐tier surface structures (AM‐E and Reg‐E), *h*_c_ of the AM‐E ranged from 37.8 to 52.0 ± 6.3 kW (m^−2^ K^−1^), 45–53% higher than the Reg‐E surfaces. For the two‐tier surface structures (AM‐EB and Reg‐EB), the *h*_c_ value of the AM‐EB surface was 55–69% higher than the Reg‐EB surface. The results indicate that the length scale and structure of the microcavities of the Reg‐E surface (Figures 2b and Figure S3b, Supporting Information) are unable to prevent droplets from completely filling the cavities. Even though the hierarchical micro/nanostructures of the Reg‐EB surfaces are able to promote droplet jumping at low supersaturation, the number of water‐filled cavities increases with increasing supersaturation resulting in high surface adhesion and inhibits droplet jumping. These results further outline the advantage of the unique cellular structures which are achievable only with AM surfaces.

Figure 4c,d shows the comparison between the condensation enhancement factor (EF) for AM and conventional Al tubes, where EF represents the *h*_c_ ratio of the enhanced tube to a plain tube exhibiting filmwise condensation at the same supersaturation. The comparison quantifies the advantage of implementing functionalized structured tubes in a condenser as compared to conventional plain tubes exhibiting filmwise condensation. Furthermore, as *h*_c_ of filmwise condensation is highly dependent on the supersaturation (*S*), to provide a fair comparison, it is important to determine EF at the same *S* for a given vapor pressure. The *h*_c_ of the filmwise condensation mode was determined from the well‐validated classical Nusselt theory.^[^ ^44^ ^]^ For the AM‐EB surface with two‐tier nanostructures, the EF values ranged from 4.79 to 5.95, corresponding to a 479–595% enhancement in *h*_c_ when compared to filmwise condensation. For the AM‐E single‐tier cellular structures, high EF values ranging from 3.22 to 4.70 were achieved.

To provide an evaluation of the durability of the AM two‐tier nanostructured surface, we performed condensation experiments at high supersaturation and in pure vapor conditions on the AM‐EB tube sample over a period of seven days. On each day, one experiment was carried out at high vapor pressure (6.5 kPa) for a duration of ≈2 h. We emphasize that pure steam and high vapor pressure condensation represent more stringent conditions for durability tests when compared to condensation in the presence of air. This is because condensation rate in pure steam is several orders of magnitude higher when compared to condensation in the presence of air. This condensation rate increases the droplet nucleation rate, growth rate, and departure frequency which not only increases the stresses exerted by the droplets on the coating, but also the possibility of coating blistering and delamination. After each experiment, the chamber along with the tube sample were left in air to dry overnight. We were unable to prolong the test duration beyond 2 h due to the limited capacity of the vapor generator. Figure S12 (Supporting Information) shows time‐lapse images of condensation on the AM‐EB sample over the span of 7 days (totaling 14 h) demonstrating the dominant jumping‐droplet condensation mode with a negligible density of pinned droplets for the entire test duration.

Figure 4e illustrates the performance of the AM‐EB surface when compared to other superhydrophobic surfaces reported by other investigators on jumping droplet enhanced condensation. The best surface is selected from each of the referenced articles and the reported heat transfer coefficients (*h*_c_) are plotted. We note that in the cited references, *h*_c_ values were obtained with respect to subcooling (Δ*T*). Hence, we have also plotted our results in terms of Δ*T*. Figure 4e clearly shows that despite exhibiting high *h*_c_ at low Δ*T* (which also corresponds to low supersaturation), past structures are unable to sustain the same condensation performance with increasing Δ*T*.^[^ ^9^, ^11^, ^14^, ^45^, ^46^, ^47^, ^48^, ^49^ ^]^ For Δ*T* > 5 °C, our AM‐EB structures exhibited the best thermal performance due to their excellent antiflooding characteristics. It is also worth noting that the benchmarked existing structures were either fabricated from copper or silicon wafers, not aluminum. Despite more than a decade of intensive research, enabling jumping droplet condensation on aluminum materials in pure vapor and high supersaturation conditions remains challenging and has not been demonstrated.^[^ ^50^ ^]^ To the best of our knowledge, our work represents the first demonstration of sustainable jumping droplet condensation in pure vapor, high supersaturation conditions on aluminum materials, which is only achievable using our AM surface structuring method. Consequently, highly efficient thermal devices may no longer be limited to heavy and expensive copper alloys as nanostructured aluminum alloys can now cater for lightweight applications for industries including aviation and aeronautics, automotive, electronics packaging, and electric and unmanned aerial vehicles.

**Figure 5:**
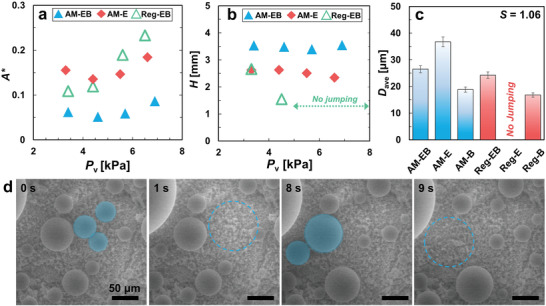
Analysis of condensed droplet dynamics. a) Average area coverage by pinned droplets (A*) and b) average droplet jumping height (*H*) under pure vapor condition for 3.3 kPa < *P*_v_ < 7.0 kPa. Measurements only for the surfaces exhibiting droplet jumping (i.e., AM‐EB‐, AM‐E, and Reg‐EB) have been shown. AM‐EB exhibited the lowest A* and highest *H*. c) Average droplet departure diameter (*D*_ave_) of all AM and conventional Al samples measured using an environmental scanning electron microscope (ESEM) at 800 ± 50 Pa, *T*_w_ = 3.0 ± 0.5 ^○^C and *S* = 1.06. d) Exemplary time‐lapse ESEM images during water vapor condensation showing droplet jumping on the AM‐E surface at *S* = 1.06. For additional ESEM images, see Figure S13 in the Supporting Information. The measurement uncertainty of A* ranged from 3.2% to 8.3%, while the uncertainty of *H* ranged from 1.0% to 2.3%, and the uncertainty of *D*_ave_ ranged from 1.5% to 2.4%.

To obtain further insights into the nanostructure‐defined droplet morphology on the AM and conventional Al‐alloy superhydrophobic surfaces, we performed high speed optical imaging analysis to quantify the average surface area covered by pinned droplets (A*), droplet jumping height (*H*) and droplet departure diameter (*D*_ave_). The condensation process on each tube was captured using a high‐speed camera (Phantom VEO 640L) at 1000–1500 frames per second (fps) and a field of view of 4.5 × 3.4 cm. Hence, the projected view of 4.5 cm length of each tube was obtained for analysis. The steam saturation pressure (*P*_v_) was varied between 3.3 and 7 kPa. For each pressure increment, the system was allowed to stabilize for about 10 min before high‐speed videos were recorded (see Figure S14 and Video S1 in the Supporting Information for the exemplary images and high‐speed video). The value of A* was calculated by taking the ratio of the total projected area of the pinned droplets on a tube to the projected area of the tube surface, where the latter was fixed at 566 mm^2^ in this analysis. A pinned droplet was defined as a droplet with diameter larger than 100 µm. Previous studies have shown that droplets of such length scale have significantly high thermal resistance and contribute to less than 5% of the condensation heat transferred from the surface.^[^ ^51^ ^]^ **Figure** **5**a shows A* of the sample tubes which exhibit droplet jumping (AM‐EB, AM‐E and Reg‐EB). At low vapor pressure (*P*_v_ ≤ 4 kPa), even though all three tubes exhibit droplet jumping, the AM‐EB tube had a significantly smaller A* and number of pinned droplets. In contrast, even though the A* values of Reg‐EB were smaller than AM‐E for *P*_v_ ≤ 4 kPa, its surface quickly flooded at *P*_v_ > 4 kPa, leading to a large increase in A*.

Using a similar approach, the average droplet jumping height (*H*) for AM‐EB, AM‐E, and Reg‐EB were determined. The jumping droplet phenomena on the various tubes and at different vapor pressures were captured using the high‐speed camera at 1500 frames s^−1^. Side views of the tubes similar to those shown in Figure 3 and Figure S11 (Supporting Information) were taken. To ensure measurement accuracy, the average jumping height was determined by tracing the droplet trajectory only for the droplets departing from the tube top surface. To ensure statistical significance, up to 15 droplets were measured for each pressure and the average values were presented.

As two or more neighboring droplets coalesce, excess surface energy (Δ*E*) is converted into translational kinetic energy (*E_k_*). During coalescence and departure, energy is dissipated to overcome the droplet‐surface interfacial adhesion. Therefore, higher *H* is indicative of lower surface adhesion. Figure 5b shows that the AM‐EB tube exhibited the highest *H* of ≈3.5 mm. The AM‐E tube showed slightly lower *H* of 2.4–2.7 mm. The jumping height of the AM‐EB sample was higher than the AM‐E sample, supporting our hypothesis that the two‐tier boehmitized‐cellular nanostructures of the AM‐EB surface are the enabling factor for droplet‐surface adhesion reduction (Figure S9b, Supporting Information).

The droplet departure diameter has direct influence on the thermal and wetting performance of the surface. A smaller departure diameter results in higher droplet renewal frequency and lower thermal resistance. To obtain precise measurement of jumping droplets (<50 µm), we conducted additional experiments using environmental scanning electron microscopy (ESEM, Figure 5c). Figure 5d shows exemplary time‐lapse images of the droplet growth dynamics, coalescence, and departure on the AM‐E surface (see Figure S13 in the Supporting Information for ESEM time‐lapse images of all AM and conventional Al surfaces). For the ESEM studies, *P*_v_ was fixed at 800 ± 50 Pa, *T*_w_ = 3.0 ± 0.5 ^○^C and *S* = 1.06, with average droplet departure diameter (*D*_ave_) determined by averaging the droplet sizes of all jumping events within a 5 min steady‐state condensation interval (Figure 5c). Despite the AM‐B and Reg‐B not being able to prevent flooding at high supersaturation (see Video S1 in the Supporting Information), at low supersaturation, good droplet repellency and small departure diameters (*D*_ave_ < 20 µm) were observed (see Figure S13c,f in the Supporting Information), suggesting that the AM‐B and Reg‐B surfaces are promising for applications where supersaturations are low and noncondensable gases are present such as water harvesting^[^ ^1^ ^]^ and dehumidification.^[^ ^9^ ^]^ Past studies have shown that a larger wetted area beneath the partially wetting droplet results in larger droplet departure diameters.^[^ ^8^, ^52^, ^53^ ^]^ Even though the AM‐E cellular structures have the ability to prevent flooding at high supersaturations, their larger cellular cavity size results in slightly larger jumping droplet departure diameter (*D*_ave_ = 37 µm for AM‐E and *D*_ave_ = 26 µm for AM‐EB) as compared to AM‐B and Reg‐B at low supersaturation (*S* = 1.06). A similar ESEM approach was used to determine the droplet jumping frequency (see Section S7 of the Supporting Information for method). Based on the high resolution images taken, the jumping frequencies of the AM‐E, AM‐EB and Reg‐EB surfaces were found to be (0.74 ± 0.1) × 10^6^ m^−2^ s^−1^, (0.98 ± 0.1) × 10^6^ m^−2^ s^−1^ and (0.78 ± 0.1) × 10^6^ m^−2^ s^−1^, respectively, with AM‐EB demonstrating the highest jumping frequency. This result further verified the low droplet‐surface adhesion and excellent antiflooding characteristics of AM‐EB, which not only facilitates droplet detachment but also reduces the pinned droplet sites.

## 3 Fabrication of a Codesigned Nanostructured Additively Manufactured Condenser

**Figure 6:**
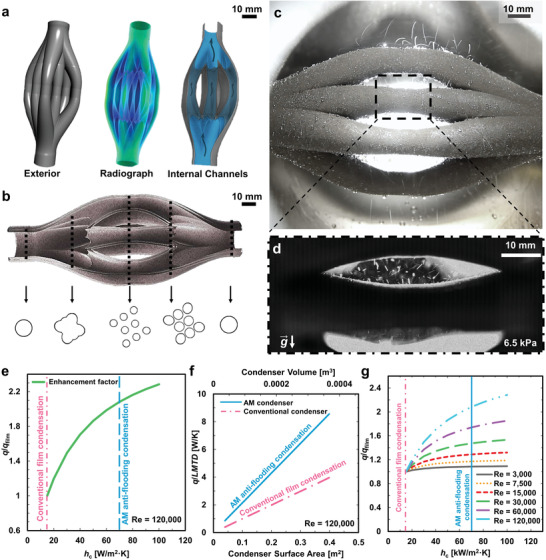
Characterization and jumping droplet condensation on an AM condenser. a) X‐ray computed tomography (CT) scans of the AM heat exchanger exterior, radiograph showing interior, and cross section showing internal flow direction (coolant false colored in blue). b) CT scan obtained heat exchanger cross‐sections along the length of the tube. c) Jumping‐droplet condensation on the two‐tier nanostructured AM heat exchanger at *P*_v_ = 6.5 kPa. d) Close‐up view of droplet jumping phenomenon taken using a high‐speed camera. e) Enhancement factor (*q*/*q*_film_) of a condenser with different condensation heat transfer coefficient (*h*_c_) as compared to a condenser exhibiting filmwise condensation. The tube O.D. and I.D. are fixed at 12.7 and 10.7 mm, respectively, and the internal water flow is maintained at Re = 120 000. f) Overall thermal performance of AM heat exchanger demonstrating the need for smaller surface area and tube volume as compared to conventional heat exchanger. The internal water flow is maintained at Re = 120 000. g) Heat exchanger enhancement factor at different *h*_c_ and Re showing the advantage of AM antiflooding condensation.

For practical implementation, scalability of the structured surface using the widely expanded design space offered by AM is crucial to overcome physical constraints and improve thermal performance.^[^ ^54^, ^55^ ^]^ To demonstrate the scalability of the present AM nanostructuring method and its applicability in enabling surface superhydrophobicity of complex geometries, we fabricated a codesigned and highly compact multitube heat exchanger by using SLM and further enhanced its surface by implementing the two‐tier (boehmitized cellular) nanostructures. **Figure** **6**a,b shows X‐ray computed tomography (CT) images of the physical two‐tier nanostructured AM heat exchanger which consists of larger circular water inlet and outlet sections of outer diameter (O.D.) 25.4 mm with eight smaller tube branches at the middle with minimum O.D. of 11.0 mm. The heat exchanger layout is typical of a shell‐and‐tube condenser with cooling water flowing through the internal flow channels and steam condensing on the external surface. The converging/diverging sections which connect the larger tubes to eight smaller tubes are at ≈45° from the centerline to avoid overhang, enabling the efficient additive manufacturing of the entire heat exchanger vertically without supports. Furthermore, the monolithic manufacturing of the entire heat exchanger eliminates joining processes such as welding and reduces water‐side pressure drop by the integration of smoother fluidic transitions and bends. Figure 6b shows CT scans of the internal surface and cross sections along the length of the AM heat exchanger prior to surface treatment. Figure 6b shows that the surface is free from macrodefects and the internal flow channels are cleared of residual powder. Water condensing on the AM superhydrophobic heat exchanger at the vapor pressure of ≈6.5 kPa is shown in Figure 6c. A close‐up image of the top and middle row tubes taken using high‐speed imaging is shown in Figure 6d. Droplet‐jumping condensation, similar to that observed on the AM‐EB tube (Figure 3c) was observed on the AM heat exchanger. Due to the intense droplet jumping process and proximity of the smaller tubes, droplets departing from one tube were observed to impact adjacent tubes and, in the process, assisted in the removal of locally pinned droplets, further increasing droplet renewal frequency and improving heat exchanger performance.

The overall thermal performance of a condenser depends on the: 1) external condensation heat transfer coefficient (*h*_c_) and its associated thermal resistance, 2) tube wall conduction thermal resistance, and 3) internal water‐side heat transfer coefficient (*h*_w_) and its associated thermal resistance given by Equation (S4) in the Supporting Information. To demonstrate the advantage of the AM condenser over a conventional tube bank condenser exhibiting filmwise condensation, we further quantified their performance using condensation experimental data obtained in Figure 4, existing correlations for internal flows given by Equation S5 (Supporting Information) and Nusselt's equation^[^ ^44^ ^]^ for external filmwise condensation. Taking a constant water flow through each condenser tube with *Re* = 120 000 for instance, it is evident from Figure 6e that the present AM two‐tier nanostructures have the potential to exhibit enhancement factor (*q*/*q*_film_) of 2 as compared to a conventional falling film condenser. Here, it should be noted that the enhancement factor (*q*/*q*_film_) reported in Figure 6e,g considers both the water‐side and vapor‐side thermal resistances and differs from the EF presented in Figure 4c,d which only considers the vapor‐side thermal resistance. Consequently, in accordance with Equation (S5) (Supporting Information) and depicted in Figure 6f, for both condensers to have the same performance (*q*/LMTD), a smaller heat transfer area and hence reduced amount of material is required for the AM two‐tier nanostructured condenser, making the AM condenser more compact and light weight.

As with all condensers, the coolant water‐side single‐phase thermal performance is usually lower than the vapor‐side two‐phase condensation performance. If the water‐side thermal resistance is too large, it may dominate the overall performance of the condenser. This effect is presented in Figure 6g. With the increase in coolant flow rate (which increases the water‐side heat transfer coefficient), a more significant improvement of the condenser performance can be realized by achieving antiflooding condensation using the AM nanostructured surface. This analysis also points to the importance of enhancing the water‐side thermal performance. Passive enhancement methods can be achieved by introducing internal structures, often at the millimeter or submillimeter size, in the flow channels, which can be challenging to fabricate by conventional manufacturing due to accessibility issues and manufacturing limitations. However, with the wide design freedom provided by AM, new enhanced features can be easily integrated inside the coolant flow channel of the AM condenser in a single manufacturing process to further increase overall performance.^[^ ^27^, ^28^, ^56^, ^57^, ^58^ ^]^

## 4 Corrosion Resistance

**Figure 7:**
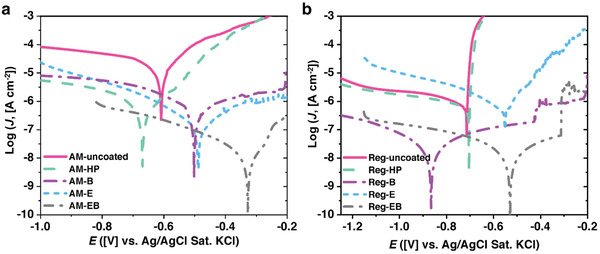
Tafel polarization curves. a) Polarization curves for all AM surfaces and b) all conventional Al surfaces. The uncertainty in potential is ± 1 mV ± 0.2% of the reading and the uncertainty in current is 0.75% of the reading. The working solution is 0.5 m NaCl.

Corrosion characterization is important to provide an estimation of the durability and long‐term stability of the developed AM surfaces. We conducted polarization tests on the 1) unaltered AM (AM‐uncoated) and conventional Al surfaces (Reg‐uncoated), 2) silanized hydrophobic AM and conventional Al surfaces (AM‐HP and Reg‐HP), and 3) all the structured superhydrophobic AM and superhydrophobic conventional Al surfaces (AM‐B, AM‐E, AM‐EB, Reg‐B, Reg‐E, and Reg‐EB). The results of the polarization tests are presented in **Figure** **7** (see Section S8 of the Supporting Information for details of experimental setup and procedures). To further quantify the corrosion resistance performance of the surfaces, we used Tafel extrapolation technique to extract the corrosion currents from the polarization curves. The results are summarized in **Table** **4** where the corrosion rates (*ψ*) are calculated by*ψ* = 3.302*I*_corr_EW*ρ*, where *I*_corr_ is the current density in units of [µA cm^−2^], EW is the equivalent weight which is defined as the atomic weight in units of [g] divided by the valency, and *ρ* is the metal density in units of [g cm^−3^]. The corrosion rate unit is in [µm year^−1^].

**Table 4:** Tafel extrapolation fitting parameters for all AM and conventional Al surfaces obtained from polarization tests in 0.5 M NaCl solution

Surface	*E*_corr_ [V]	*I*_corr_ [µA cm^−2^]	*b*_a_ [mV dec^−1^]	*b*_c_ [mV dec^−1^]	*ψ* [µm year^−1^]
AM‐uncoated	−0.609	38.6	216.7	1346.5	424.85
AM‐HP	−0.669	1.55	326	450	17.06
AM‐B	−0.502	0.62	358	127	6.82
AM‐E	−0.498	0.156	288	78.9	1.71
AM‐EB	−0.327	0.0152	140	190	0.167
Reg‐uncoated	−0.714	0.64	44	303	7.04
Reg‐HP	−0.704	0.58	30.8	436	6.38
Reg‐B	−0.866	0.073	861	574	0.803
Reg‐E	−0.553	0.879	110	410	9.67
Reg‐EB	−0.53	0.014	209	221	0.154

Figure 7 and Table 4 illustrate that the AM‐EB and Reg‐EB exhibit the smallest corrosion currents which indicates the highest corrosion resistance. The high corrosion resistance is attributed to the synergistic effect of the boehmite layer, and the additional porous structures introduced by etching. A single tier boehmite layer acts as a barrier to corrosive ions and protects the underlying surface.^[^ ^59^ ^]^ Furthermore, the air pockets inside the micro/nanostructures of the superhydrophobic surfaces provides additional protection by decreasing the available active area for corrosion. The trapped air within the structures prevents the corrosive ions from attacking the underlying surface.^[^ ^60^, ^61^ ^]^ As shown in the SEM image of the AM‐E (Figure 2a), the surface structures have a cellular well‐ordered shape resulting in more porous structures capable of trapping more air inside the pores when compared to the boehmite only nanostructured surface. However, when the additional boehmite protective layer is added to the AM‐E, it plays two significant roles. First, it acts as an additional protective oxide layer to the surface which keeps the underlying porous structures safe from the corrosive ions increasing the long‐term stability of the structures. Second, it forms the hierarchical structures which decrease water adhesion as shown in our condensation experiments, limiting the concentration of the corrosive ions near the surface.

It is also evident from Figure 7 and Table 4 that AM‐B has the highest corrosion current, hence lowest corrosion resistance among the structured AM surfaces. This poor corrosion resistance is due to the presence of un‐melted non‐Al powder which prevents the formation of boehmite on the AM surface as shown in the SEM images of Figure S6 (Supporting Information). The Reg‐E sample has the lowest corrosion resistance among all the structured conventional Al surfaces. This is due to the weak bonding of the silanes to the etched Al surface due to the reduction in oxides and hydroxyl groups stemming from the surface etching process.^[^ ^62^ ^]^ During the corrosion tests, as soon as silane is removed from the rough Reg‐E surface, the surface becomes exposed directly to the NaCl solution. The corrosion current (*I*_corr_) is calculated by normalizing the measured current (µm) by sample base projected area (cm^2^). However, due to the larger surface roughness of Reg‐E (rms of ±10.7 µm, see Figure 2i), this resulted in a larger area being exposed to the corrosive solution. This in turn caused the corrosion current of Reg‐E to be higher even when compared to the nonstructured Al surface which has significantly lower surface roughness (rms of ±3.3 µm, see Figure 2h). The weaker bond between the HTMS layer and the Reg‐E surface is further confirmed as the surface loses superhydrophobicity after the corrosion test is conducted. However, the HTMS bond with the AM and AM‐E surfaces is stronger due to the higher silicon content along with its native oxide on the AM surfaces (see Figure 2j–n and Tables 1 and 2). Conversely, due to the nonuniform boehmitization of the AM surface as mentioned before, AM‐B has poorer anticorrosion performance compared to the AM‐E surface. It should also be noted that unlike Reg‐E, both of these surfaces remained superhydrophobic after the corrosion test. The stronger bonding of the HTMS to the AM surface is further verified by comparing the polarization curves of the AM‐uncoated, AM‐HP, Reg‐uncoated and Reg‐HP samples. While polarization curves of Reg‐uncoated and Reg‐HP surfaces almost overlap each other, the polarization curves of the AM‐HP surface exhibits approximately two orders of magnitude smaller corrosion current than the AM‐uncoated surface. The AM‐uncoated surface has much higher corrosion current and poorer anticorrosion performance than the Reg‐uncoated surface due to denser grain boundaries. However, even a monolayer of a hydrophobic coating (HTMS) makes the AM‐HP corrosion current comparable to the Reg‐HP Al surface as illustrated in Figure 7. This result indicates that corrosion prevention of the as‐fabricated AM surface can be easily achieved by hydrophobic HTMS coating.

## 5 Discussion

The findings reported here have important implications for the development of highly efficient devices using AM. More broadly, our demonstration of fundamental insights into the surface structuring of AM‐relevant materials, coupled with the wide design space offered by AM, has the potential to radically enhance two‐phase heat transfer in multiple sectors, including boiling,^[^ ^63^ ^]^ evaporation,^[^ ^64^ ^]^ condensation,^[^ ^53^ ^]^ frosting,^[^ ^65^ ^]^ spray cooling,^[^ ^66^ ^]^ fouling,^[^ ^67^ ^]^ and many more. We clearly showcase this paradigm shift with a focus on heat exchangers for pure steam condensation processes at high supersaturation where current state‐of‐the‐art methods focus on enhancement of filmwise condensation by passive techniques such as finned surfaces.^[^ ^55^ ^]^ Due to the limitation of our experimental facilities, our experiments were limited to maximum supersaturation of *S* = 2.0. At the highest tested supersaturation, the two‐tier nanostructured AM surface demonstrated excellent droplet jumping, with no sign of flooding when compared to conventional superhydrophobic surfaces. This observed behavior indicates that the AM superhydrophobic surfaces have the potential for further exploitation at even higher heat fluxes to overcome classical barriers identified in the droplet jumping condensation field.

Despite being a powder bed fusion technology, our work along with others^[^ ^28^, ^33^ ^]^ have shown that AM parts produced by SLM are robust and can operate in high pressure environments. These characteristics along with our developed nanostructuring technique provide opportunities for a wider range of dropwise condensation applications of AM surfaces involving low‐surface‐tension fluids commonly found in chemical plants,^[^ ^68^ ^]^ biomass production^[^ ^69^ ^]^ and refrigeration systems.^[^ ^5^ ^]^ To promote dropwise condensation of low‐surface‐tension fluids, structured superhydrophobic surfaces are infused with a thin layer of low energy lubricant.^[^ ^70^, ^71^ ^]^ Also known as lubricant infused surfaces (LISs), past works have demonstrated the potential of repelling ethanol and hydrocarbons owing to their low contact angle hysteresis and surface energy. Our development of scalable methods to fabricate structured superhydrophobic AM surfaces thus paves the way for future work to develop AM LISs, extending LISs beyond conventionally manufactured metallic materials. Durability of LISs resulting from the depletion of lubricant is a concern, limiting their long‐term application. Even though there has been recent success in prolonging the life span of LISs to beyond 8 months when condensing ethanol,^[^ ^72^ ^]^ further improving the longevity of the surfaces is required. In the future, it would be interesting to determine AM LIS performance in retaining the lubricant and identifying how the AM structure morphology can be further optimized to limit lubricant depletion.

The mechanical robustness of a superhydrophobic surface to retain its nonwetting ability under harsh operating environments remains a concern. A design strategy to improve the surface resilience to wear can be realized by housing the fragile nanostructures within a protective, high hardness, structural frame of larger length scale.^[^ ^73^ ^]^ As the cell walls of the AM‐EB surface consist of Si‐rich phase which has excellent hardness, they can serve as the protective frame for the second tier boehmite nanostructures within cells. This characteristic of the AM structures provides further opportunities for an abrasion resistant surface to be developed. Improvements in durability of low surface energy nanoscale promoter coatings, on the other hand, can be realized by increasing the coating interfacial adhesion to prevent delamination in wet environments. Recent work has demonstrated that the utilization of lipid‐like bilayer coatings have the potential to exhibit such durability, hence, prolonging jumping‐droplet condensation for more than 8000 h.^[^ ^74^ ^]^ Therefore, combining our AM nanostructuring strategy with lipid‐like bilayer coatings presents a feasible approach towards realizing jumping‐droplet enhanced condensation for industrial scale application and can be investigated in the future.

The work presented here represents a comprehensive study to identify both mechanisms and methods to enable surface structuring of Al‐alloys used in AM. In the future, it would be interesting to develop similar design guidelines for alternate AM materials including Copper‐alloys (such as CuCr1Zr), stainless steel (such as 316L), Titanium‐alloys (such as Ti6Al4V) and Cobalt‐alloys (such as CoCrMo). The development of these alternate materials is motivated by the inability to use AlSi10Mg materials for selected applications such as ultrahigh heat flux electronics cooling requiring higher thermal conductivity alloys such as CuCr1Zr, or seawater condensers, in which seawater is utilized as the coolant and flows through the internal tube channels,^[^ ^59^ ^]^ requiring excellent corrosion resistance facilitated by alloys such as 316L.

In addition to alternate materials, future work is needed to identify codesign methodologies to achieve the more synergistic integration of surface structuring and AM design methods. Our demonstration here of a shell and tube condenser focused on developing an integration and demonstration platform to enable surface structuring scale‐up. Future work is needed to identify key design paradigms which are enabled by the developed structuring technique, and how they can be synergistically integrated with AM design to develop ultrahigh power density two‐phase heat transfer components. As shown in Figure 6c,d, the intense droplet jumping on the AM condenser result in the removal of locally pinned droplets as jumping‐droplets from adjacent tubes impact on these pinned droplets. Hence, a possible approach to maximize condensation rate would be to optimize the tube arrangement and separation to take advantage of this droplet removal mechanism. In addition to multitube condensers identified here, additional applications include shell and tube boilers, as well as flow condensation and boiling, where surface structures have been shown to significantly enhance two phase heat transfer, and integration with AM techniques will significantly reduce manufacturing complexity and eliminate thermal interfaces which is the key to performance enhancement.

As compared to untreated metallic surfaces which are intrinsically hydrophilic, suitably designed superhydrophobic AM surfaces can also be ideal for delaying frost growth (i.e., in heat pump applications) by efficient removal of condensed droplets via coalescence‐induced jumping.^[^ ^3^, ^62^, ^75^ ^]^ Thus, the combination of AM design and our micro/nanostructuring technique not only enables enhanced power density of heat exchangers, but the surface hydrophobicity also reduces frost formation^[^ ^3^ ^]^ and allows the heat exchangers to operate efficiently even in cold climates, thus, extending their application range. Beyond thermal devices, superhydrophobicity is a key requirement for many applications, including self‐cleaning, antibacterial function, reduced particulate adhesion, and many others. Furthermore, the developed nanostructuring approach in combination with AM need not enable jumping droplet condensation. Nanostructured AM condensers can be fabricated and made superhydrophilic to promote filmwise condensation by enhancing condensate drainage, and promote porous condensation and film thinning.^[^ ^76^, ^77^ ^]^ The developed methods presented here open the door for the synergistic integration of metal AM with multifunctional surfaces for these nonthermal uses in structural and biological engineering.

## 6 Conclusions

In summary, we demonstrate superior two‐phase heat transfer performance through the synergistic combination of additive manufacturing (AM) and surface structuring enabled by highly scalable and inexpensive nanostructuring methods. Using condensation as the demonstration platform, we show that rationally designed and nanostructured superhydrophobic surfaces made with AM have superior performance when compared to conventional manufactured Al‐alloy surfaces, as demonstrated via the ultrastable antiflooding performance and coalescence‐induced jumping at high supersaturations. Our two‐tier nanostructured AM surface showed a 6X and 2X higher steam condensation heat transfer coefficient when compared to state‐of‐the‐art filmwise and dropwise condensation surfaces, respectively. Through well‐controlled condensation experiments, high resolution visualization, and droplet motion analysis, we conclusively demonstrate that our developed two‐tier structures for AM surfaces are able to prevent lateral spreading of droplets and reduce droplet‐surface adhesion. By leveraging the excellent design freedom of AM with the exceptional scalability of our nanostructuring method, we design and fabricate a complex two‐tier nanostructured superhydrophobic AM heat exchanger to demonstrate superior performance at high condensation supersaturations. Further, we compared the corrosion performance of all unaltered and structured AM and regular Al surfaces by potentiodynamic polarization characterization and showed the enhanced corrosion protection of the two‐tier nanostructured superhydrophobic surfaces. This work not only develops a facile and scalable method to achieve nanostructured AM Al‐alloy surfaces, but it also provides surface structuring guidelines for AM materials by opening up opportunities for further advancing metal AM for a plethora of applications.

## Conflict of Interest

The authors declare no conflict of interest.

## Author Contributions

J.Y.H. and K.F.R. contributed equally to this work. N.M., T.N.W., K.C.L., and W.P.K. supervised the project. N.M., J.Y.H., and K.F.R. conceived the idea for the work. Sample fabrication, sample characterization, condensation experiments, ESEM and data analysis were carried out by J.Y.H. and K.F.R. S.K. conducted the electrochemical corrosion experiments and data analysis. S.S. developed the condensation chamber. J.Y.H., K.F.R., S.K., and N.M. wrote the manuscript with inputs from all coauthors.

## Supporting information

Supporting Information

Supplemental Video 1

Supplemental Video 2

## Data Availability

The data that supports the findings of this study are available from the corresponding author upon reasonable request.
