# Supplementary material for: Ultrascalable Surface Structuring Strategy of Metal Additively Manufactured Materials for Enhanced Condensation
Source: Adv Sci (Weinh). 2022 Jul 3;9(24):2104454. doi: 10.1002/advs.202104454 (PMC9404399; doi:10.1002/advs.202104454)
Supplement: Supplementary file 1 — Supporting Information [file ADVS-9-2104454-s001.pdf]

ADVANCED SCIENCE
Open Access

Supporting Information

for *Adv. Sci.*, DOI 10.1002/advs.202104454

Ultrascalable Surface Structuring Strategy of Metal Additively Manufactured Materials for Enhanced Condensation

Jin Yao Ho, Kazi Fazle Rabbi, Siavash Khodakarami, Soumyadip Sett, Teck Neng Wong, Kai Choong Leong, William P King and Nenad Miljkovic**

Supporting Information for

Ultra-Scalable Surface Structuring Strategy of Metal Additively Manufactured Materials for Enhanced Condensation

Jin Yao Ho[a,b,+], *Kazi.Fazle Rabbi*[a,+], *Siavash Khodakarami*[a], *Soumyadip Sett*[a], *Teck Neng Wong*[b,*], *Kai Choong Leong*[b], *William P. King*[a,c,d], *Nenad Miljkovic*[a,c,d,e,*]

[a] *Department of Mechanical Science and Engineering, University of Illinois at Urbana-Champaign, Urbana, IL, 61801, USA*

[b] *Singapore Centre for 3D Printing, School of Mechanical and Aerospace Engineering, Nanyang Technological University, 50 Nanyang Avenue, Singapore 639798, Republic of Singapore*

[c] *Department of Electrical and Computer Engineering, University of Illinois at Urbana-Champaign, Urbana, IL, 61801, USA*

[d] *Materials Research Laboratory, University of Illinois at Urbana-Champaign, Urbana, IL, 61801, USA*

[e] *International Institute for Carbon Neutral Energy Research (WPI-I2CNER), Kyushu University, 744 Moto-oka, Nishi-ku, Fukuoka, 819-0395, Japan*

[+] *Equal contribution*

*Corresponding Authors E-mail: nmiljkov@illinois.edu (N. Miljkovic),
mtnwong@ntu.edu.sg (T.N. Wong)

S1. High Speed Videos

The videos were captured with a high-speed camera (Phantom VEO 640L) at 1000-1500 frames per second (fps) and were played back at 10 fps. The exposure time was kept at 150-200 µs. The cooling water flow rate was approximately 30.6 ± 0.2 L/min. The resolution of each video is 1280 × 960 pixels, which is 35.2 µm/pixel representing the dimensions of 4.5 × 3.4 cm.

S2. Fabrication Details

Additive Manufacturing Procedure: The additive manufactured samples used in this study were fabricated from AlSi10Mg powder of 20 to 63 μm distribution size using the selective laser melting (SLM), an AM technique for producing metallic parts. SLM is a powder bed fusion technology which utilizes a Gaussian distributed Yb:YAG laser sources. The laser has a maximum power of 400 W and was used to selectively melt the base metallic powder. As the powder melts, ultra-thin molten pools (≈ 50 μm) are formed which then quickly cools and solidifies, forming a dense solid layer. By successively melting and fusing the powder bed layer-by-layer three-dimensional parts are formed. The remaining un-melted powder can then be processed and reused. In this study, the SLM280HL AM facility from SLM Solutions was used to fabricate the samples where the laser power of 200 W, scanning speed of 1300 mm/s and hatch spacing of 0.08 mm were selected based on our previous experience of fabricating bulk components with good structural integrity[1-5]. In this work, all samples are fabricated in vertical orientation. Following the SLM fabrication, the as-fabricated tubes were removed from the base Al plate using a wire electrical discharge machining (EDM). We have taken multiple factors into consideration before selecting the optimum selective laser melting (SLM) process parameters for the fabrication of our AM surfaces. The quality of the bulk material produced by the process depends mainly on the laser power and laser scanning speed. When the laser power is too high or scanning speed is too low, the melt pool volume becomes too large resulting in poor printing accuracy. Furthermore, high laser power may also result in deep penetration into the previous layer causing substrate delamination and the printing process to fail. If the laser power is too low or scanning speed is too high, the energy from the laser source may not be sufficient to melt and fuse the base powder and bond the adjacent layers. This will result in a balling phenomenon where the solid region becomes discontinuous. A detailed parametric study was performed in previous study[6] for laser power between 170 and 200 W and scanning speed of 0.2 m/s to 1.5 m/s. It was found that the laser power of 200 W and scanning speeds of 1.3 to 1.5 m/s give the best structural integrity and lowest porosity of the bulk printed material[6]. Furthermore, these laser parameters have produced parts with better mechanical properties (i.e., ultimate tensile strength and hardness) than conventionally cast aluminum[7]. This is mainly due to the Si phase at the cell boundaries which works as precipitates to hinder the dislocation movements during deformation and improves material strength. More importantly, these laser parameters were also chosen for our past studies[1-5] where the fabricated parts exhibited good thermal properties and were able to withstand extremely high-pressure operation without failure, which are important factors for this study.

Cleaning Procedure: The AM and regular Al tubes were thoroughly cleaned prior to the surface treatment process. All tubes were sealed using a female ½” stainless steel Swagelok tube fitting on both ends and capped with a ½” stainless steel Swagelok nut to ensure the internal surfaces of the tubes were not affected by the surface treatment process. Subsequently, the tubes were separately immersed in acetone (Sigma-Aldrich, CAS No. 64-64-1), ethanol (Sigma-Aldrich, CAS No. 64-17-5) and isopropyl alcohol (Sigma-Aldrich, CAS No. 67-63-0) each and ultrasonicated for 10 min. Thereafter, the tubes were rinsed with deionized water (Sigma-Aldrich, CAS No. 7732-18-5) and dried with nitrogen stream.

Chemical Etching Procedure: To produce the first-tier nanostructures on an AM tube, the as-fabricated tube (after cleaning) was dipped into a diluted 2.0 M hydrochloric acid (Sigma-Aldrich, CAS No. 7647-01-0) solution for 2.5 min at room temperature. Following the etching process, the tubes were further ultrasonicated for 20 min to remove residues and dried with clean nitrogen gas. On the other hand, to produce the first-tier microscale roughness on a conventional Al alloy tubes, the as-purchased Al tube (after the cleaning) was dipped into a 2.0 M hydrochloric acid (HCl) solution for 15 min at room temperature[8]. The AM and regular Al tubes that underwent this chemical etching procedure have been named AM-E and Reg-E, respectively. It should be noted that, after etching, as the reagent etches only the top surface layer of the material, the mechanical properties of the material are not expected to deviate significantly. This is further verified by our chamber condensation experiments (see Section S4) where the AM-E and Reg-E tubes were able to sustain long term operation under high vacuum pressure environment, at high internal coolant flow rate (~ 30 LPM) without exhibiting any sign of leakage or degradation in material integrity.

Boehmitization Procedure: The nanoscale roughness in the form of boehmite or aluminum oxy-hydroxide (Al(O)OH) structures were formed by immersing the cleaned AM or Reg-Al tubes into a hot pool of DI water for 30 mins. Throughout the immersion process, the DI water was maintained at the temperature of 95°C ± 2°C. The hot water causes a self-limiting reaction at the aluminum surface forming a thin layer of boehmite of approximately ~300 nm. The AM and regular Al tubes that underwent this boehmitization procedure are named AM-B and Reg-B, respectively.

To produce the two-tier nanostructures on AM tube, the AM tube that underwent the HCl etching was further boehmitized based on the procedure described above, resulting in boehmite formation on the first-tier nanostructures. The AM tube that underwent both etching and boehmization procedures is named AM-EB.

Similarly, to produce the two-tier micro/nanostructures on regular Al tube, the Al tube that underwent the HCl etching was further boehmitized based on procedure described above, resulting in boehmite formation on the first-tier microstructures. The regular Al tube that underwent both etching and boehmitization procedures is named Reg-EB.

Silanization Procedure: The tube samples were functionalized using atmospheric chemical vapor deposition of a conformal layer of heptadecaflouro-tetrahydrodecyl trimethoxy silane (HTMS, Gelest, CAS #83048-65-1). The tubes were placed in a sealed glass container, along with 5% v/v of HTMS-toluene. The container was placed inside a furnace, maintained at 90°C, for 3 hours. This process resulted in the deposition of a monolayer of HTMS on the tube surfaces making them superhydrophobic.

The illustration of the single-tier and two-tier nanostructures are shown in Fig. S1. In addition, all tube samples fabricated based on the abovementioned procedures are shown in Fig. S2. Finally, additional small flat plates (1” × 1”) were fabricated using the same procedures described above for surface characterization, to measure the contact angle using microgoniometer and to determine the condensate droplet departure size using ESEM.

S3. Surface Characterization

Scanning Electron Microscope (SEM)/ Focused Ion Beam (FIB): SEM analysis of the fabricated samples was conducted with a Hitachi S4800 scanning electron microscope. The cross-sectional (FIB) images were taken with Thermo Scios2 Dual-Beam.

Figure S3 shows the cross sectional (FIB) images of the micro/nanostructures of all the samples. The SEM images of these samples at the same magnifications are shown in Figure S5. Exemplary measurements of the nanostructure dimensions (boehmite) are shown in Figure S4. In addition, SEM surface characterization of AM-B revealed the presence of un-melted granular particles on its surface. Figure S6's SEM images show the absence of boehmite formation on some of these particles because of their low Al content. It should be noted that chemical etching process helps to remove these un-melted granular particles and as a result AM-E and AM-EB surfaces are free from such defects.

Contact Angle Measurement: The surface wettability of all samples was characterized immediately after the silanization procedure. Contact angle measurements of ≈ 100 nL droplets on all samples were performed using a micro-goniometer (MCA-3, Kyowa Interface Science Co. LTD.). A piezoelectric dispenser was set 5-10 mm above the sample surface and the dispenser would dispense microscale droplets on the surface, allowing droplets to accumulate into a larger droplet to measure apparent advancing contact angle (θ_a). To measure apparent receding contact angle (θ_r), the dispenser was turned off, allowing the water droplet to evaporate. At least three measurements were performed on spatially varying location of each sample surface and at each location an average of 10 sampling points were obtained. All contact angle data were analyzed using the image processing software (FAMAS software, Kyowa Interface Science Co., LTD.) with an in-built circle fitting algorithm. The maximum measurement errors of θ_a, θ_r and $\Delta\theta$ are ±3°, ±3° and ±4.2°, respectively.

Energy-dispersive X-ray Spectroscopy (EDS): EDS analysis of the samples was conducted using the FEI Quanta FEG 450 ESEM. To study the chemical composition on the Al alloy surfaces, wide band EDS scanning was performed on eight random spots of each AM and conventional Al-6061 samples before and after the chemical etching process.

X-ray photoelectron spectroscopy (XPS): XPS data was obtained using a Kratos Analytical Axis Ultra with a monochromatic Al Kα X-ray source. The size of the source beam was 2 mm × 2 mm, and the size of the analyzed region was 0.3 mm × 0.7 mm. The instrument was maintained at a pressure of 10^{-7} Pa during the experiments. The spectra data interpretation and analysis has been conducted with Casa XPS software (Casa Software Ltd.).

Confocal microscopy: To determine the surface topography and roughness of the samples before and after HCl etching, the confocal microscopy analysis was performed to determine the roughness of the samples. The Keyence VK-X1000 3D Laser Scanning Confocal Microscope was used to obtain the high-resolution confocal microscopy images. Here, it is emphasized that the main purpose of performing the confocal microscopy analysis is to provide an overview of the samples' surface roughness (at the macro-level) before and after chemical treatment. For the

determination of submicron scale topology, atomic force microscopy (AFM) is usually performed. However, performing AFM on etched Al surfaces is very challenging due to the large surface roughness. Hence, to provide insights on the submicron scale topology such as the nanocell size, depth and wall thickness, we performed both SEM and FIB on the test samples. While SEM analysis provides information of the structure size and thickness from the top view, the structure depth is determined from FIB measurements.

Verification of cellular grain structures on AM surfaces: To verify the AM samples also consist of nano-sized cellular structures, we performed Keller's etch on our AM surface, a process widely used to reveal the grain boundaries of AM samples. The surface of an AM sample was first polished to a mirror-like finish using sandpaper of grit size 1000, 2000, 3000, 5000 and 7000, respectively. Thereafter, the surface is cleaned by sonicating in acetone, ethanol and IPA for 10 min each. Once the surface is cleaned and air dried, it is immersed in the Keller's reagent, consisting of 2.5% HNO_3, 1.5% HCl, 1% HF and 95% water, for 30s. Figure S7 shows the SEM images of the AM surface after etching with Keller reagent. Clearly, cellular-like structures can be observed. The slight difference between the HCl etched cellular structures (Fig. 2a) and the Keller reagent etched cellular structures (Fig. S7) results from the combination of HF and HNO_3 in Keller reagent reacting with Si-rich cell walls. Nonetheless, from this investigation, the presence of cellular structures with Si-rich walls and Al-rich core on the AM samples has been verified. Here, we emphasize that due to the low temperature of the AM platform (~150°C), the molten pool undergoes extremely high solidification rates, leading to the epitaxial growth of crystals. Since Si and Al have different solidification temperatures, Si is rejected toward the solid-liquid interface as the molten pool cools. This reduces the Si content in the liquid. After solidification, supersaturated Al-rich cell cores are formed with the residual Si as the cell boundaries. Recent works have compared the intrinsic structures of as-fabricated AlSi10Mg of same Si concentration by conventional casting and AM method[9,10]. It is determined that the nano-cell structures are only found on AM AlSi10Mg whereas conventional casting produced AlSi10Mg does not contain such nano-cells. This confirms that the nano-cells are produced as a result of the high melting and solidification rate of the AM process

Verification of AM etching mechanism and optimized etching time for AM surfaces: To verify the etching mechanism, we performed a detailed parametric study by systematically varying the etching time to show that HCl etches only the Al-rich cell core and with increasing etching time, cellular structures with deep pores are generated. For this study, four 1" × 1" AM flat samples were prepared and each sample was etched by immersing in 2M HCl at 1 min, 1.5 min, 2.5 min and 4.0 min, respectively. The SEM images of each sample after the etching process is shown in Fig. 2(a). It is evident from this figure that the initial etching for 1 min only reveals the shallow cell structures while the cell walls start to become visible. With 1.5 min of etching, the Al-rich core begins to react with the etchant and variation in the pore depth can be observed. For 1.5 min etching time, some cells show completely removed Al-rich core while some cell cores remain partially etched. With further increase in etching time, more cellular structures with deeper cell core and Si-rich walls becomes visible. To further demonstrate that an optimized etching time has been achieved, we performed goniometric measurements to quantify the samples' wetting characteristics. Figure 2(c) shows the variation of the advancing contact angle with etch time. Note that all the samples were converted to superhydrophobic surface by

silanization prior to contact angle measurement. It can be seen that the advancing contact angle initially increases with etch time (*t*) but stabilizes at about 160° after $t \geq 2.5$ min.

Determination of optimized etching time for conventional Al surfaces: For conventional Al samples (Reg-E), we have conducted similar investigation to identify the appropriate etching time (*t*) and determined the effects of etching time on surface morphology. In this study, the etching time was varied from 1 min to 20 min. Five 1" × 1" flat Al-6061 samples were prepared, and each sample was etched by immersing in 2M HCl for 1 min, 2 min, 5 min, 10 min, 15 min and 20 min, respectively. Figure 2(b) shows the SEM images of the Al-6061 surfaces after etching. For $t \leq 1$ min, there is no change in surface morphology and the surfaces look similar to an unmodified aluminum. As *t* increases beyond 1 minute, microstructures begin to form. However, the surfaces remain non-uniformly etched and patches of un-etched regions can be seen. A uniformly etched surface was achieved only after 15 minutes of etching and further increment in etching time yielded similar surface structure morphology. To further demonstrate the optimized etching time, we performed contact angle measurements using micro-goniometry to quantify the samples' wetting characteristics. Figure 2(c) shows the variation of the advancing contact angle with etching time. Note that all the samples were converted to superhydrophobic surface by silanization prior to contact angle measurement. Figure 2(c) illustrates that the advancing contact angle increases with *t* and stabilizes after 15 minutes of etching. This confirms our optimized selection of etching time to promote superhydrophobicity for conventional Al surface.

S4. Pure Vapor Condensation Experiment

Experimental Facility: The heat transfer coefficients of the tubes are determined using the test facility shown in Fig. S8. Details of the test facility and experimental procedures have been reported in our past works[11,12] and are briefly described here. The test facility consists of an environmental chamber, vapor line and coolant line. The environment chamber, where the tube sample to be tested are housed, has an internal diameter of 0.305 m and length of 0.559 m. Both ends of the chamber are sealed with flanges. The chamber is installed with six viewports (5.08 cm diameter ports from MPF Products and 6.35 cm diameter ports from MDC Vacuum), each with diameter of about 6 cm, for visual access. Several feedthrough fittings are mounted on the parameters of the chamber so that thermocouples and resistance temperature detectors (RTDs) can be installed within the chamber to obtain the vapor and coolant temperatures. To monitor the chamber pressure, two pressure transducers (Baratron 728A and 925 MicroPirani from MKS Instruments) have been used with one transducer installed at each end of the chamber. The vapor line consists of a vapor generator (8" O.D. Standard CF Tee, Kurt J. Lesker) filled with the DI water and a stainless-steel tubing (1/2" O.D. from Swagelok) system connecting the pressure vessel to the environmental chamber. To heat up the DI water, three rope heaters (Part #AWH-101-040DP, ETS Equipment), each of 624 W, are installed on the outer surface of the vapor generator. The rope heaters are connected to a variable power transformer (Model PM-1220BE, ETS Equipment) which allows the heaters' heat rate to be controlled. A T-type thermocouple (Part #SCPSS-032, Omega) is inserted into the vapor generator to monitor the working fluid's temperature. The coolant line is a closed loop system consisting of a chiller (Part #327005091602, System III TU7 Pump, Thermo Fisher Scientific) with a build-in water pump. Cold water is supplied from the chiller through the tube sample located in the environmental chamber and the water flow rate is measured using an electromagnetic flow meter (Part #FMG93, Omega). The cold-water inlet/outlet temperatures are measured using two RTDs (Part #AT-PX1123Y-LR4S1T2T, ReoTemp) installed at the two ends of the tube sample.

Experimental Procedures: Prior to the start of the experiments, the internal walls of the condensation chamber are wiped clean with acetone and isopropyl alcohol to remove any contaminant. The vacuum pump (Model Alcatel 2005) connected to the condensation chamber is then turned on to remove any traces of non-condensable gas from the system. A liquid nitrogen cold trap (Model #TLR4X1100QF), installed just before the vacuum pump, allows moisture from the air extracted from the chamber to be removed and assisted in improving the chamber vacuum condition. During the vacuuming process, the tape heaters are concurrently turned on to heat up the DI water in the vapor generator. It should be noted that, during this process, the valves (Model #6L-LD8-BBXX, Swagelok) installed on the tube connecting the vapor generator and condensation chamber are kept closed. The DI water is degassed by heating it to 100°C for more than 10 minutes. When the chamber pressure is below 50 Pa, the chiller is turned on to allow cold water to circulate through the internal channel of the sample tube. During the experiments, the inlet cold water temperature ($T_{w,in}$) is maintained between 7°C and 8°C and the flow rate ranges between 20 ± 0.2 to 32 ± 0.2 L/min which correspond to the Reynolds number (Re) of 28,000 to 45,000 and Prandtl number (Pr) of approximately 10. The experiments are commenced when the chamber pressure is below 6 ± 2 Pa. Thereafter, the valve installed on the connecting tubes between the vacuum pump and condensation chamber (Model #6L-LD8-BBXX, Swagelok) is closed and the vacuum pump is switched off. The valve on the vent port of

the vapor generator (Model #SS-8BW, Swagelok) is closed to isolate the vapor generator from ambient pressure. Finally, the valve connecting the pressure vessel and condensation chamber (Model #6L-LD8-BBXX, Swagelok) is gradually opened to achieve the required vapor pressure (P_v). In this investigation, P_v has been varied between 3.2 kPa and 7.5 kPa and the condensation heat transfer coefficients were determined at different P_v values, corresponding to different supersaturation (S).

Heat Transfer Characterization: The heat transfer coefficient is obtained by first determining the condensation heat transfer rate (q_d) of each tube sample from heat rate gained by the cold water, given by Eq. (S1). Using the log-mean-temperature-difference ($LMTD$) method, the overall thermal conductance (UA) of a tube can be computed by Eq. (S2), where $LMTD$ is obtained by Eq. (S3). On the other hand, UA consists of the condensation heat transfer coefficient (h_c), water-side heat transfer coefficient (h_w) and thermal resistance across the tube wall as shown in Eq. (S4). Using the thermal conductivity value (k) of the tube material, the inner (d_i) and outer (d_o) tube diameters and total length (L) of a tube, the thermal resistance across the tube wall can be determined. In this study, the k value of Al-6061 was taken as 167 $W/m^2 \cdot K$[13] whereas the k value of the AM material (AlSi10Mg) was taken as 124 $W/m^2 \cdot K$ based on our previous measurements[14]. The values of h_w are determined using the Petukhov correlation[15] given by Eqs (S5) and (S6). The experimental uncertainty of the heat transfer measurement is determined by propagating the instrument uncertainties shown in Table S1, based on the method described by Moffat[16]. The uncertainties of h_c are reported with each result. It is noted that in our previous studies[11], the accuracy of the experimental results was validated by performing filmwise condensation on a horizontal plain tube and the results showed good agreement with the classical Nusselt theory[17].

Finally, we noted that the difference in surface roughness mainly affects the water-side heat transfer coefficient (h_w) due to the change in friction factor (f). The average surface roughness (R_a) of the as-fabricated AM sample obtained from our confocal microscopy analysis is 7.2 μm and conventional Al-6061 is 2.6 μm. This gives the relative roughness, a ratio of R_a to inner tube diameter, of 6.7×10^{-4} and 2.4×10^{-4}, respectively. Apart from Petukhov correlation[15], the Colebrook-White correlation[18] provides a relation between the relative surface roughness and friction factor. Considering Re of 44,000, which is currently used in our experiments, it is determined that deviation in h_w between AM and conventional Al-6061, computed by Colebrook-White correlation, is only about 2.8% and about 4.3% when compared to the Petukhov correlation. The small deviation is due to the significantly smaller surface roughness when compared to the tube diameter, hence a small change in relative roughness. Due to the small difference in friction factor, the Petukhov correlation[13] is employed in this work.

$$q = c_{\mathrm{p,w}} m_{\mathrm{w}} \left(T_{\mathrm{w,out}} - T_{\mathrm{w,in}} \right) \tag{S1}$$

$$UA = \frac{q}{LMTD} \tag{S2}$$

$$LMTD = \frac{(T_{\mathrm{sat}} - T_{\mathrm{w,in}}) - (T_{\mathrm{sat}} - T_{\mathrm{w,out}})}{ln\left[\frac{(T_{\mathrm{sat}} - T_{\mathrm{w,in}})}{(T_{\mathrm{sat}} - T_{\mathrm{w,out}})}\right]} \tag{S3}$$

$$\frac{1}{UA} = \frac{1}{h_{\mathrm{c}}A_{\mathrm{o}}} + \frac{ln(d_{\mathrm{o}}/d_{\mathrm{i}})}{2\pi L k_{\mathrm{Al}}} + \frac{1}{h_{\mathrm{w}}A_{\mathrm{i}}} \tag{S4}$$

$$h_w = \left(\frac{k}{d_{\mathrm{i}}}\right)\frac{(f/8)\mathrm{RePr}}{1.07 + 12.7(f/8)^{1/2}(\mathrm{Pr}^{2/3} - 1)}\left(\frac{\mu_{\mathrm{b}}}{\mu_{\mathrm{s}}}\right)^n \tag{S5}$$

For $10^4 \le \mathrm{Re} \le 10^6, \quad 0.5 \le \mathrm{Pr} \le 200$

$$f = [0.79\ln(\mathrm{Re}) - 1.64]^{-2} \tag{S6}$$

The etched layer, boehmite layer and silane layer contribute to the wall thermal resistance of the tube. However, as the combined thickness of these nanostructures and silane monolayer is small, it has insignificant effect on the condensation heat transfers (h_c) presented in this study. To provide a quantitative analysis, we consider a conservative case where the AM-E sample consists of cellular nanostructure of thickness (δ) 1.5 μm forming on the external surface of a tube with 1 mm wall thickness (t). This would result in an additional resistance term $\delta/((\pi d_o L)(\phi k_s + (1-\phi)k_v))$ to the right-hand side of Eq. (S4), where ϕ is the solid fraction, k_s is the thermal conductivity of the nanostructures and k_v is the thermal conductivity of the void region between the nanostructures. The solid fraction is estimated to be 0.35 obtained by grayscale analysis of the SEM images as shown Fig. 2(a) using Image-J software. For a conservative estimation, k_v is considered 0, as the condensation experiment was performed under high vacuum condition. Figure S16 shows a comparison of the h_c values of AM-E with and without the effect of nanostructure thermal resistance. It is evident that this effect is negligible as the small nanostructure thickness results in an insignificant increase in thermal resistance.

Enhancement factor: The condensation enhancement factor (*EF*) for structured AM and conventional Al tubes is computed by taking the h_c ratio of the enhanced tube to a plain tube exhibiting filmwise condensation at the same supersaturation. In our previous study, we have validated the accuracy of the used test facility by running filmwise condensation experiments at different vapor pressures[11] and the test results showed good comparison with the Nusselt model. As it is experimentally challenging to replicate filmwise condensation at the same supersaturation as the enhanced tubes, the Nusselt model is used in this study to compute *EF*. It should be also emphasized that the filmwise condensation studied in Ref. [11] was performed on a conventionally produced metallic tube where its surface is smoother than the untreated AM material. For untreated AM surfaces, we have also determined their filmwise condensation performance for steam as reported in Ref. [3]. In Ref. [3], a flat AM plate instead of a tube was tested and the experimental results compared well with the values predicted by the Nusselt

filmwise model. Even though the AM surfaces are rough, as the surfaces are covered by the liquid film during filmwise condensation, the heat transfer is dominated by the liquid film resistance and the roughness has no significant effect on the condensation performance. To further verify filmwise condensation on AM surface can be predicted by the Nusselt model, we also performed additional filmwise condensation experiments of ethanol on an as-fabricated AM tube (O.D. 12.7 mm) in our test chamber of Fig. S8 for P_{sat} ranging from 6.7 to 12.6 kPa and ΔT from 11.2 to 19.1°C. Our experimental results again compared well with Nusselt film theory with all deviations lying within the experimental uncertainties.

S5. Work of Adhesion

Considering two adjacent partially wetted droplets just before coalescence as shown in Fig. S10, the work of adhesion in this state is the sum of adhesion in the wetted ($W_{\mathrm{a,wetted}}$) and non-wetted ($W_{\mathrm{a,non\text{-}wetted}}$) regions of the droplet-surface interface[19, 20, 21], given as Eq. (S7), where n denotes the number of coalescing droplet, i.e., $n = 2$. The adhesion in the non-wetted region of a droplet resulting from the area in contact between the droplet base and nanostructures can be calculated by Eq. (S8). Note that in this equation, φ represents the solid-liquid contact area (or nanostructure void fraction), σ_{lv} is the liquid-vapor interfacial surface tension, $\theta_{\mathrm{r}}^{app}$ is the droplet apparent receding contact angle and A_{b} is the droplet base area given by Eq. (S9), where R is the droplet radius. The adhesion in the wetted region due to the liquid filled cavities underneath the droplet can be expressed as Eq. (S10) where A_{p} is the projected area of the liquid filled cavities in contact with the droplet. Assuming the liquid filled cavities have the same equivalent cavity size, r_{p}, A_{p} can then be computed by Eq. (S11), where m is the number of liquid filled cavity. On the other hand, θ_{w} is the contact angle between the droplet and the condensate in the liquid filled cavity which is 0°. Hence, substituting Eqs. (8) and (10) into Eq. (S7) and taking $n = 2$ gives the final form of the work of adhesion (W_{a}) as Eq. (S12).

$$W_{\mathrm{a}} = n\left[W_{\mathrm{a,wetted}} + W_{\mathrm{a,non-wetted}}\right] \tag{S7}$$

$$W_{\mathrm{a,wetted}} = \varphi A_{\mathrm{b}}\sigma_{\mathrm{lv}}\left(1 + \cos\theta_{\mathrm{r}}^{app}\right) \tag{S8}$$

$$A_{\mathrm{b}} = \pi R^2 \sin^2\theta \tag{S9}$$

$$W_{a,non-wetted} = A_{\mathrm{p}}\sigma_{lv}(1 + cos\theta_w) \tag{S10}$$

$$A_{\mathrm{p}} = m\pi r_{\mathrm{p}}^2 \tag{S11}$$

$$W_{\mathrm{a}} = 2\sigma_{\mathrm{lv}}\left\{\varphi A_{\mathrm{b}}\left(1 + \cos\theta_{\mathrm{r}}^{app}\right) + 2A_{\mathrm{p}}\right\} \tag{S12}$$

S6. Visualization of Vapor Condensing on Tubes Samples

In addition to high-speed imaging, real time videos of steam condensation on the tube samples have also been taken. This allows wider view of the condensation process to be captured and provides a better macroscopic perspective of the droplet dynamics on the tube samples. The cooling water flow rate was approximately 30.6 ± 0.2 L/min. After steam from the boiler was introduced to the condensation chamber, the system was allowed to stabilize for about 10 minutes. Thereafter, the videos were taken with a digital single-lens reflex (SLR) camera (Canon EOS T3i) at 24 frames per second (fps) for a duration of 3 minutes. The resolution of each video is 1920 × 1080 pixels, which is 62.5 μm/pixel representing the dimensions of 12.0 × 6.8 cm. For each video, a still image of the condensation phenomenon at the time stamp of 1.5 minutes was extracted. Figures 3 and S11 show a comparison of the steady state condensation of water on AM-EB, AM-E, AM-B, Reg-EB, Reg-E and Reg-B for vapor pressures ranging from 3.2 to 7.4 kPa and illustrations of nanodroplets growth dynamics in cellular structures in AM-E and two-tier nanostructures in AM-EB.

S7. Environmental Scanning Electron Microscope (ESEM)

The FEI Quanta FEG 450 ESEM has been used to obtain the Environmental Scanning Electron Microscopy (ESEM) images of microdroplets condensing on the AM and conventional Al surfaces. For this study, the vapor pressure (P_v) has been fixed at 800 ± 50 Pa. A Peltier stage is first used to cool the sample for about 10-15 min until it reaches thermodynamic equilibrium with the surrounding vapor. To initialize condensation, the sample is further cooled to the temperature of 3.0 ± 0.5°C, which corresponds to the supersaturation (S) of 1.06. Visualization of the droplet departure diameter, presented in Fig. 5(d) of the manuscript, is performed using a flat sample holder to obtain top view images of the condensation process and the beam potential is limited to 10 kV to avoid heating effects. Condensation on the sample surface is recorded when the droplets grow sufficiently large and begin coalescing with the neighboring droplets. The video is captured at 1 fps and played back at 1 fps and the field of view is 424 × 360 μm. Figure S13 shows the exemplary time lapse of condensation on all AM and conventional Al surfaces obtained using the ESEM.

The ESEM images of vapor condensation on all AM and conventional Al surfaces were analyzed to determine the average droplet jumping diameter and droplet departure frequency. Detailed analysis of the average droplet jumping diameter is provided in the manuscript. To obtain the number of droplet jumping event, condensation experiments were performed in the ESEM and images were recorded over a 5 min steady-state condensation interval with a field of view of 424 × 360 μm. Based on the high resolution images taken, the jumping frequencies of the AM-E, AM-EB and Reg-EB were found to be (0.74 ± 0.1) × 10^6 $m^{-2} \cdot s^{-1}$, (0.98 ± 0.1) × 10^6 $m^{-2} \cdot s^{-1}$ and (0.78 ± 0.1) × 10^6 $m^{-2} \cdot s^{-1}$, respectively.

S8. Corrosion resistance tests

The potentiodynamic polarization was conducted to characterize the corrosion resistance of the samples. Using a potentiostat (Reference 600+, Gamry Instruments) in a sodium chloride (NaCl) solution consisting of 0.5 M NaCl. A three-electrode scheme was used for the electrochemical cell. An Ag/AgCl Sat. KCl was used as the reference electrode and graphite as counter electrode. A 2.6 cm^2 area from middle of each sample was tested and the whole setup was kept inside a Faraday cage to prevent electrical interference. A detailed photograph of the entire setup is shown in Fig. S15. In this study, flat samples of 1” × 1” in size were used. Each sample was immersed in the solution for 1 hour and the open circuit potential test was done prior to starting the experiment. The potential range was set to -0.5 to 0.5 V versus corrosion potential and the scan rate was 1 mV/s.

S9. Condensation Heat Transfer Model

Following the classical dropwise condensation heat transfer model[22,23], we computed the condensation heat transfer coefficient (h_c) by Eqns. (S13)-(S17). In Eq. (S13), $r_{\min}$ and r_d represent the critical droplet nucleation radius and average droplet departure diameter given by Eqns. (S14) and (S15). The symbol r_{e} is the droplet radius when coalescence with adjacent droplets begins and is related to the nucleation density (N_{s}) shown in Eq. (S16). The symbols $n(r)$ and $N(r)$ denote the droplet size distribution in the isolated droplet growth and coalescence dominated growth regimes, respectively and can be determined by the well-established population balance theory[24] and model by Le Fevre and Rose[25]. Finally, the heat transfer rate of a droplet (q_{d}) can be determined by thermal resistances analysis, from the condensation surface to the surrounding vapor, giving Eq. (S17). In this equation, k_w, k_{s} and k_{coat} are the water, solid and HTMS thermal conductivities, δ_{s} and δ_{coat} are the solid and HTMC thicknesses and Φ is the solid void fraction. As an estimation, the values of k_w, k_{s}, k_{coat}, δ_{s}, δ_{coat} and Φ are obtained from Ref. [26].

Figure S17 shows the heat transfer coefficient determined by Eqns. (S13)-(S17) for r_d ranging from 5 μm to 100 μm and N_{s} ranging from 10^{10} to 10^{14}, respectively. In this work, the heat transfer coefficient of AM-EB (57-69 kW/m^2·K) is slightly lower when compared to jumping-droplet condensation heat transfer coefficient of ~100 kW/m^2·K reported in other works such as Ref. [26] could likely be due to the difference in (1) average droplet departure radius (r_d) and (2) base area normalized nucleation site density (N_{s}). It can be seen that both r_d and N_{s} strongly influence h_c. From our ESEM analysis, r_d is approximately 13-15 μm which is slightly larger than r_d reported in Ref. [25]. The large droplet departure radius produces additional droplet thermal resistance which reduces h_c. Furthermore, for r_d~15 μm and h_c 57-69 kW/m^2·K (as reported in our work), Fig. S17 gives N_{s} to between 10^{10} and 10^{11} for AM-EB. To achieve larger h_c, $N_{\mathrm{s}} > 10^{11}$ is required.

The droplet departure radius is largely dependent on the wetted area beneath the partially wetting droplets. As the cellular cavity size is relatively large, this could have resulted in larger r_d of AM-EB when compared to nanostructures in other works. On the other hand, N_{s} could be influenced by the surface area of the nanostructures. Nanostructures of smaller length-scales and larger density lead to larger surface area for droplet nucleation which increase N_{s}.

$$h_c = \frac{q''}{\Delta T} = \frac{\int_{r_{\min}}^{r_{\mathrm{e}}} q_{\mathrm{d}}(r)n(r)dr + \int_{r_{\mathrm{e}}}^{r_d} q_{\mathrm{d}}(r)N(r)dr}{\Delta T} \quad \text{(S13)}$$

$$r_{\min} = \frac{2T_{\mathrm{sat}}\sigma}{h_{\mathrm{fg}}\rho_{\mathrm{l}}\Delta T}, \quad \text{(S14)}$$

$$r_d = r_{\max}/1.13 \quad \text{(S15)}$$

$$r_{\mathrm{e}} = 1/4\sqrt{N_{\mathrm{s}}} \quad \text{(S16)}$$

$$q_{\mathrm{d}}(r)=\frac{\pi r^2\Delta T(1-r_{\min}/r)}{\frac{1}{2h_{\mathrm{i}}(1-\cos\theta_{\mathrm{a}})}+\frac{r\theta_{\mathrm{a}}}{4k_w\sin\theta_{\mathrm{a}}}+\frac{1}{k_{\mathrm{coat}}\sin^2\theta_{\mathrm{a}}}\left(\frac{\Phi k_{\mathrm{s}}}{\delta_{\mathrm{s}}k_{\mathrm{coat}}+\delta_{\mathrm{coat}}k_{\mathrm{s}}}+\frac{(1-\Phi)k_w}{\delta_{\mathrm{s}}k_{\mathrm{coat}}+\delta_{\mathrm{coat}}k_w}\right)^{-1}} \quad \text{(S17)}$$

Supplementary figures and tables

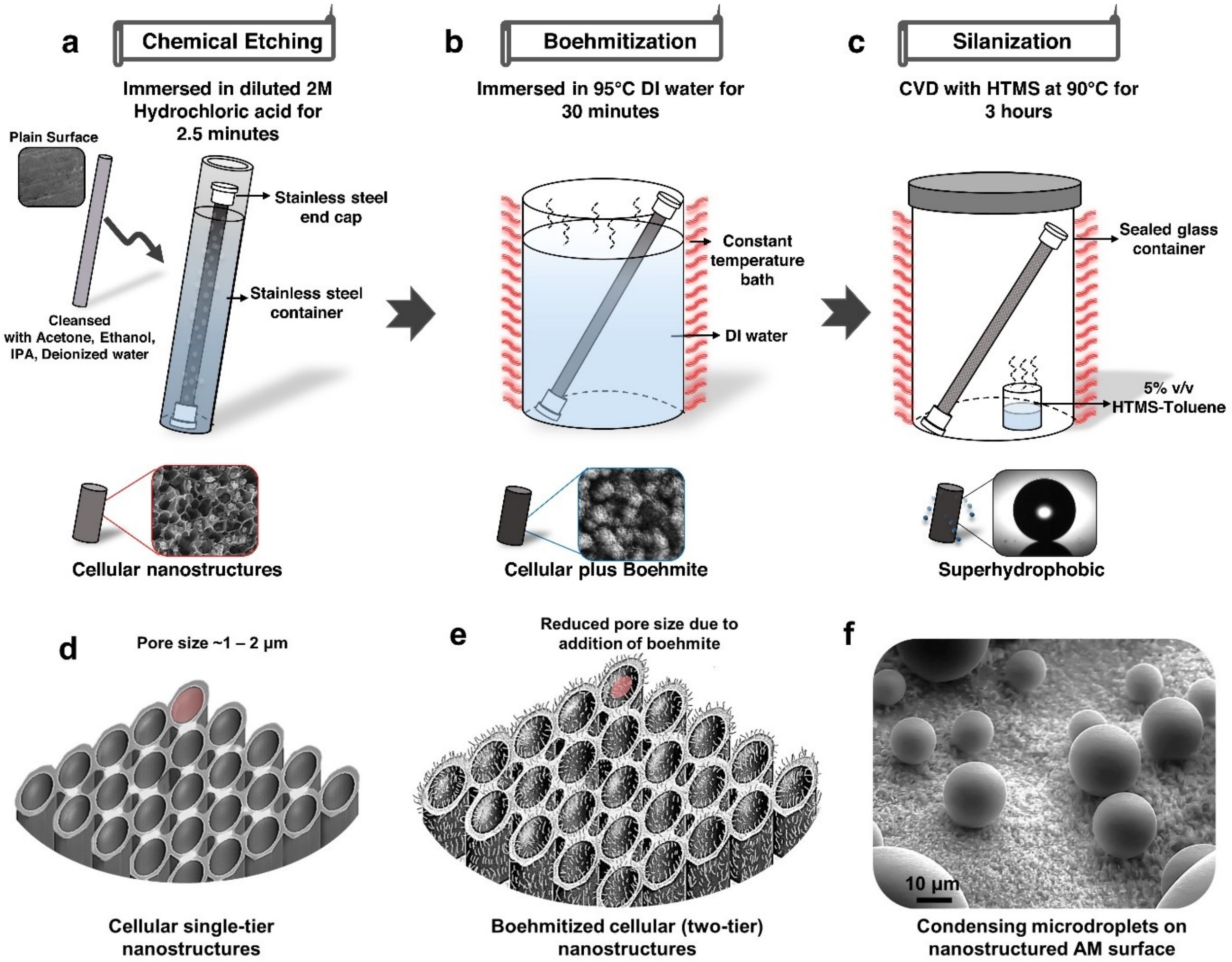

Figure S1. Schematic showing surface structure fabrication process (a) chemical etching, (b) boehmitization and (c) silanization process steps used to produce superhydrophobic two-tier nanostructures on AM tubes. Schematics of the (d) single-tier cellular nanostructure morphology on AM-E after chemical etching and (e) two-tier morphology on AM-EB after additional boehmitization illustrating the reduction of pore size due to the formation of boehmite. (f) Exemplary environmental scanning electron microscopy (ESEM) image showing condensing water microdroplets suspended on superhydrophobic nanostructured AM-E surface. Schematics are not to scale.

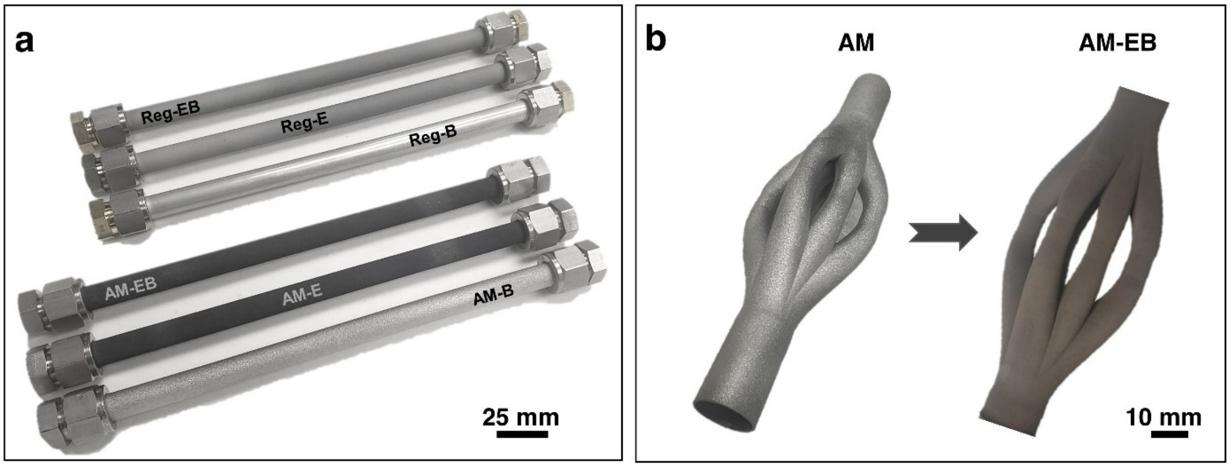

Figure S2. (a) Image of fabricated tube samples illustrating scalability of the fabrication process. Each tube has overall length of 10.5” (266.7 mm) and OD 0.5” (or 12.7 mm). Top three tubes are made of commercially available Al-6061 whereas bottom three tubes are AlSi10Mg fabricated by additive manufacturing. (b) Image of additively manufactured AM heat exchanger before and after two-tier (cellular-boehmite) nanostructuring.

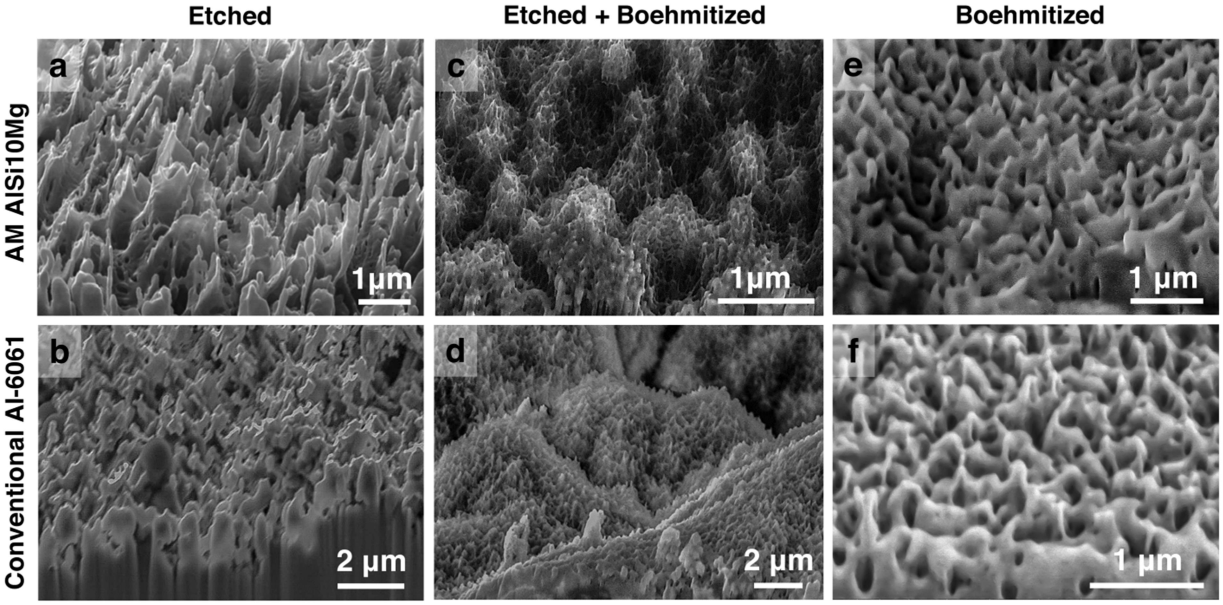

Figure S3. Scanning electron microscopy (SEM) images of the cross section obtained using focused ion beam (FIB) milling showing side view of (a) AM-E, (b) Reg-E, (c) AM-EB, (d) Reg-EB, (e) AM-B, and (f) Reg-B structures.

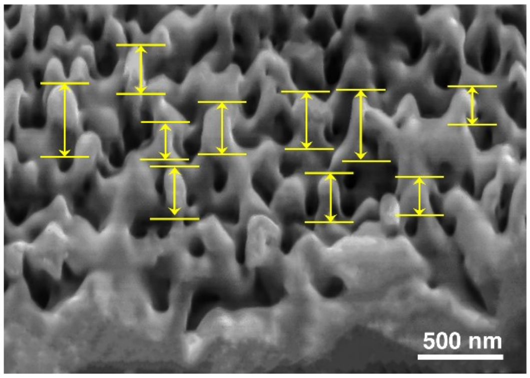

Figure S4. SEM image of the FIB cross section showing side view of boehmite structures. Height of boehmite structures were measured from the structure tip to the platelet intersect, marked with yellow lines. Approximately 20 measurements were taken, and the average value was found to be approximately 300 nm.

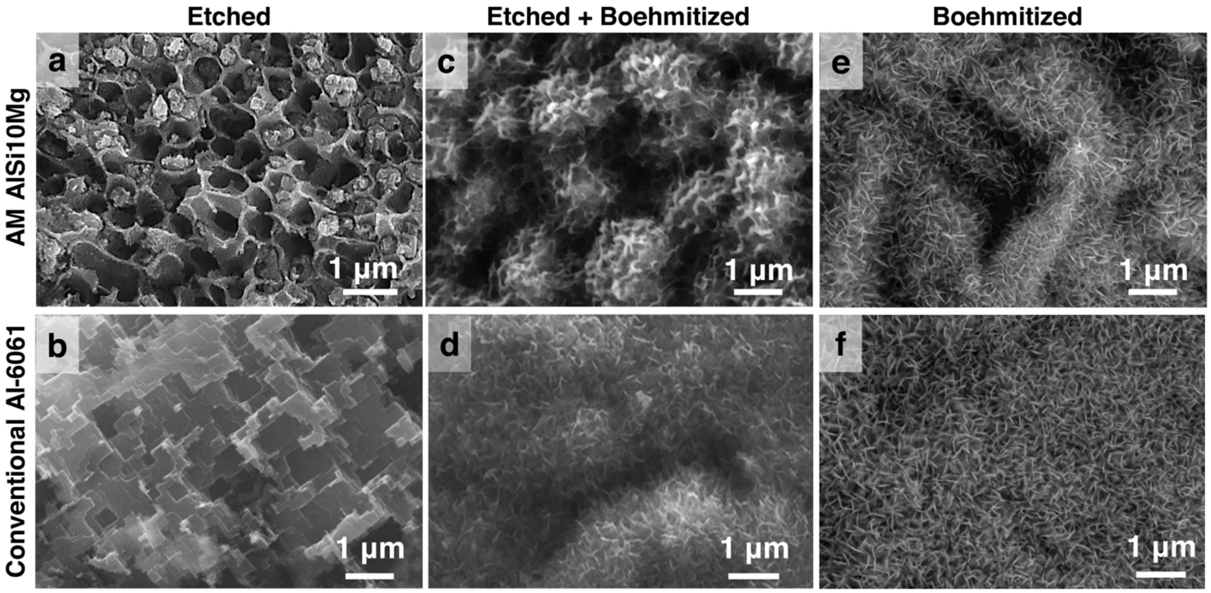

Figure S5. Top view SEM images of micro/nanostructure on (a) additively manufactured (AM) etched surface (AM-E), (b) conventional etched surface (Reg-E), (c) AM etched and boehmitized surface (AM-EB), (d) conventional etched and boehmitized surface (Reg-EB), (e) AM boehmite surface (AM-B), and (f) regular boehmite surface (Reg-B) at the same SEM magnification with scale bar of 1 μm.

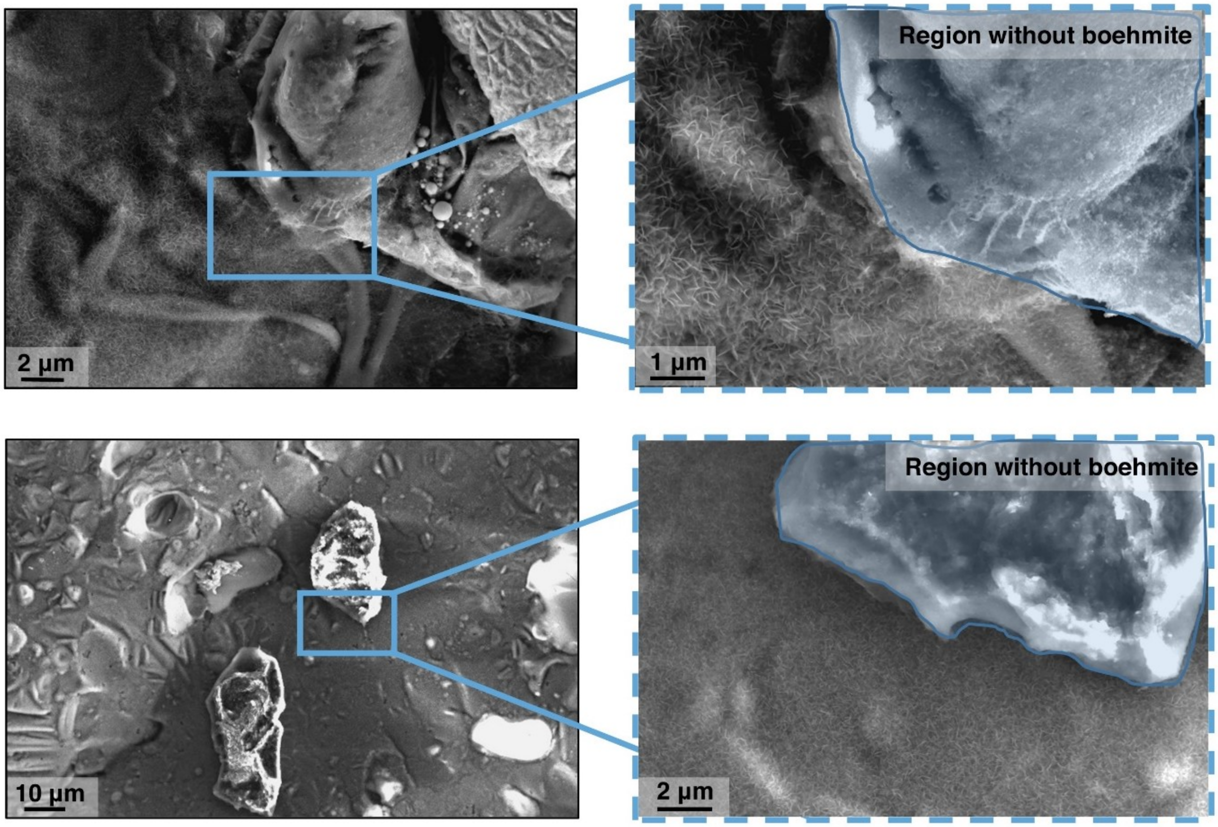

Figure S6. SEM images showing un-melted granular particles on the AM-B surfaces without boehmite formation.

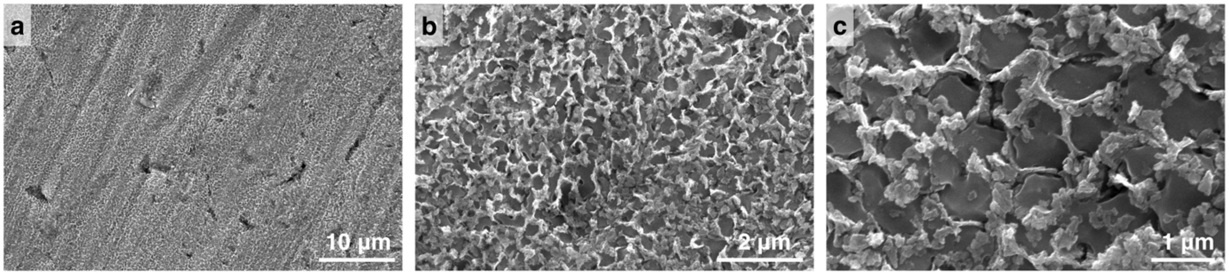

Figure S7. SEM images of AM grain structures after Keller's etch with scale bar at (a) 10 µm, (b) 2 µm and (c) 1 µm. This grain structure study clearly shows the presence of cellular-like structures.

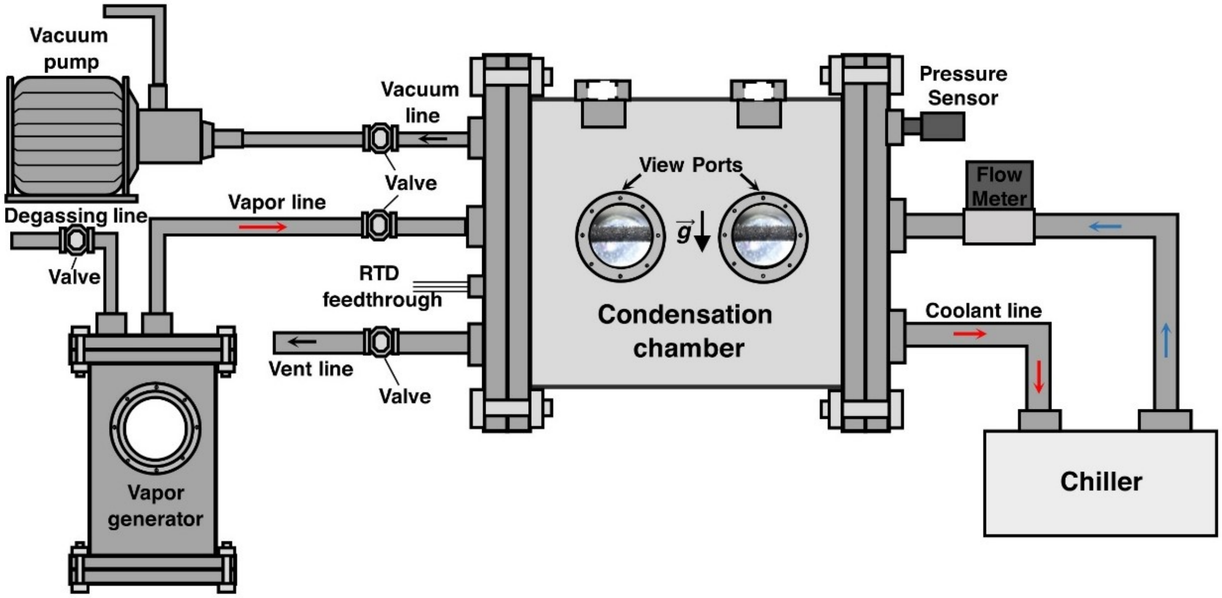

Figure S8. Schematic of test facility employed for pure vapor condensation heat transfer measurements.

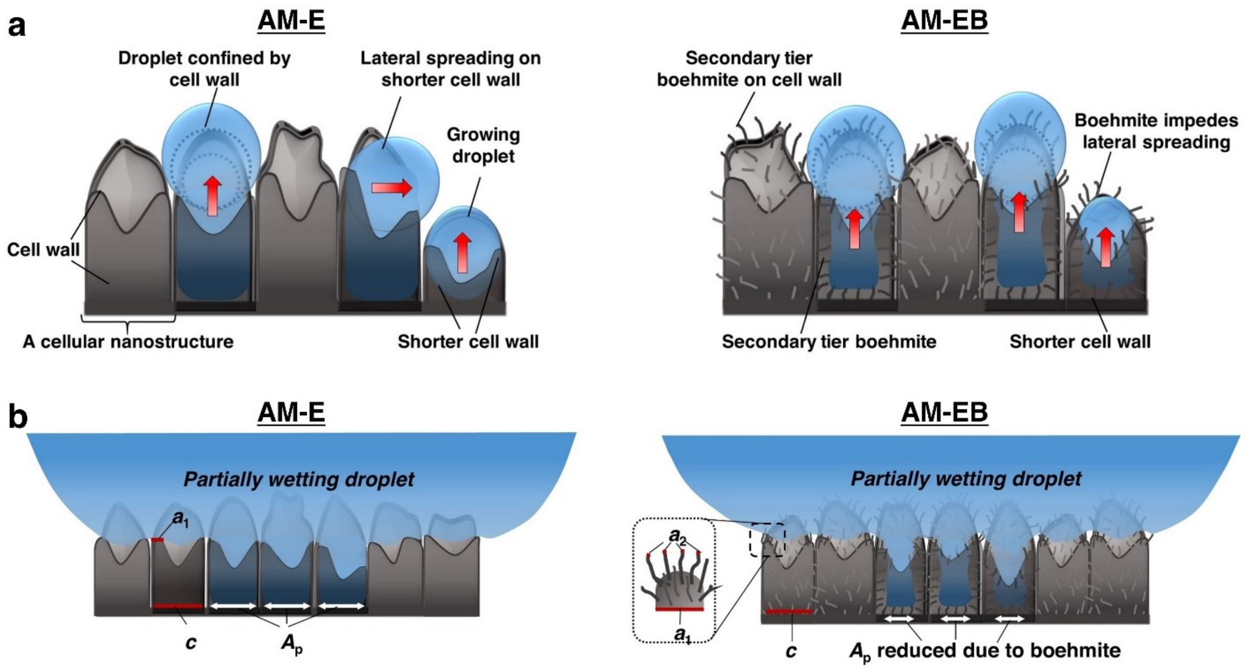

Figure S9. Schematics of (a) nanodroplet growth dynamics in cellular structures on AM-E and two-tier (cellular-boehmite) nanostructures on AM-EB surface, with AM-EB demonstrating higher energy barrier confining droplet growth within structure cavities; (b) partially wetting micro-droplets on AM-E and AM-EB surfaces with AM-EB exhibiting lower adhesion due to reduced contact area in the wetting and non-wetting regions.

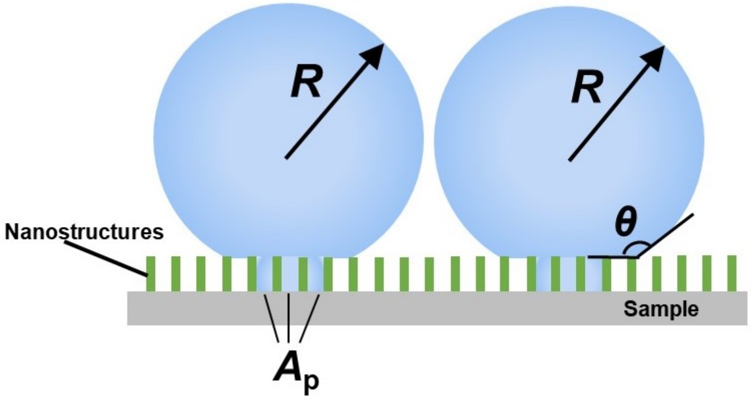

Figure S10. Simplified schematic showing two adjacent partially wetting droplets on nanostructured surface just before coalescence.

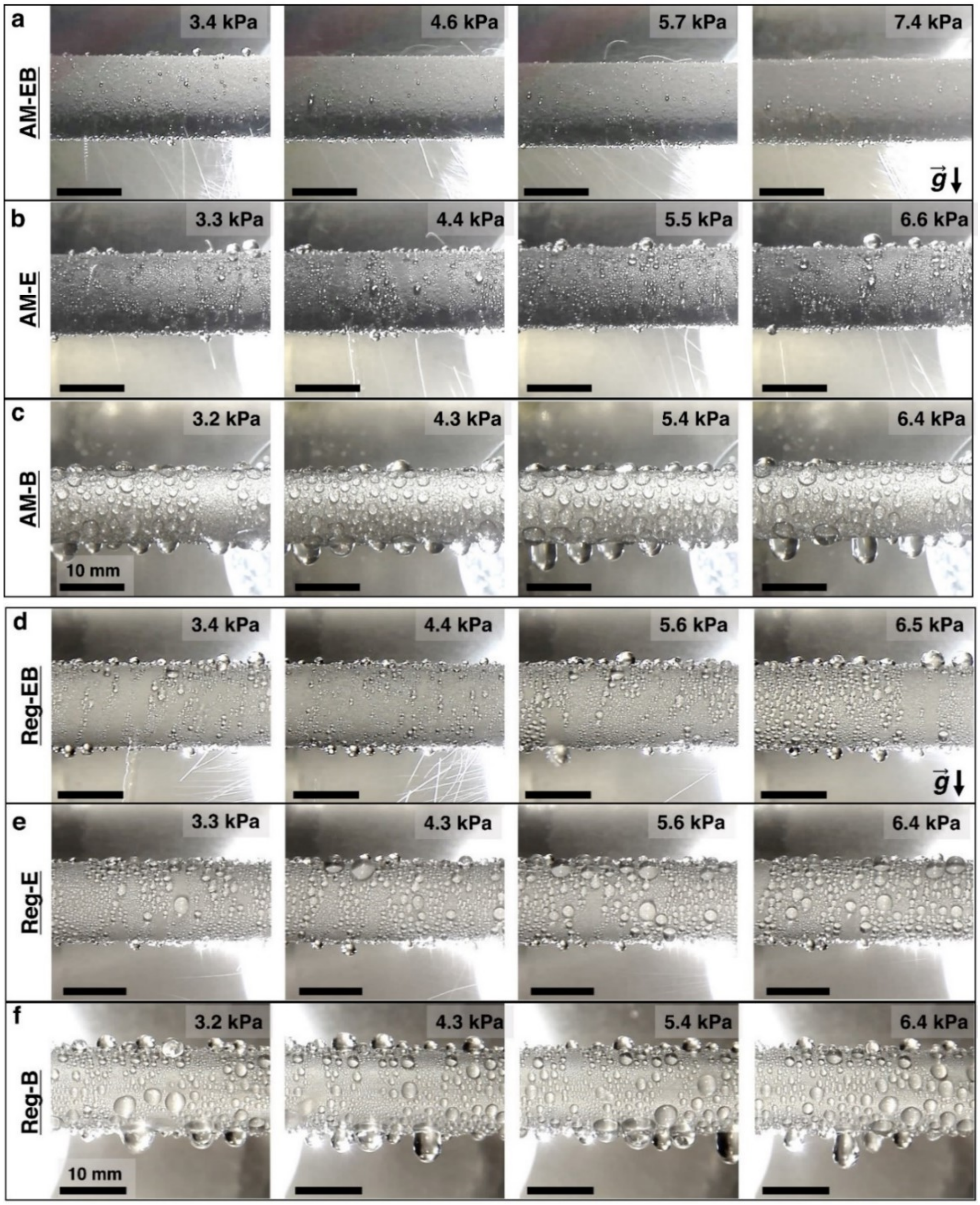

Figure S11. Images of steam condensing on (a) AM-EB at P_v = 3.4 kPa (S = 1.4), 4.6 kPa (S = 1.5), 5.7 kPa (S = 1.6) and 7.4 kPa (S = 1.8), showing highly mobile droplets leading to intense droplet jumping; (b) AM-E at P_v of 3.3 kPa (S = 1.4), 4.4 kPa (S = 1.5), 5.5 kPa (S = 1.6) and 6.6 kPa (S = 1.7), showing combination of droplet jumping and droplets rolling off the surface; (c) AM-E at P_v of 3.2 kPa (S = 1.6), 4.3 kPa (S = 1.7), 5.4 kPa (S = 1.8) and 6.4 kPa (S = 1.9), showing surface flooding and formation of highly pinned Wenzel droplets; (d) Reg-EB at P_v of 3.4 kPa (S = 1.4), 4.4 kPa (S = 1.5), 5.6 kPa (S = 1.6) and 6.5 kPa (S = 1.7), showing combination of droplet jumping and droplets rolling off the surface at low P_v and dropwise condensation with droplets rolling off the surface at higher P_v; (e) Reg-E at P_v of 3.3 kPa (S = 1.5), 4.3 kPa (S = 1.6), 5.6 kPa (S = 1.7) and 6.4 kPa (S = 1.8), showing highly pinned Wenzel droplets; (f) Reg-B at P_v of 3.2 kPa (S = 1.6), 4.3 kPa (S = 1.7), 5.4 kPa (S = 1.8) and 6.4 kPa (S = 1.9), showing highly pinned Wenzel droplets.

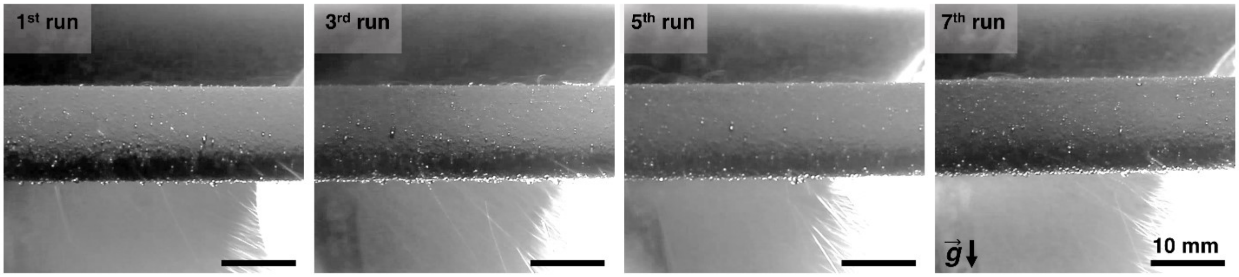

Figure S12. Time-lapse images of steam condensation on AM-EB for 14 hours over a duration of 7 days with vapor pressure (P_v) at approximately 6.5 kPa and inlet coolant water temperature (T_{in}) of 8°C. For each day, experiment was carried out for approximately 2 hr. Images show sustainable dominant jumping-droplet, anti-flooding condensation even after 7th experimental run.

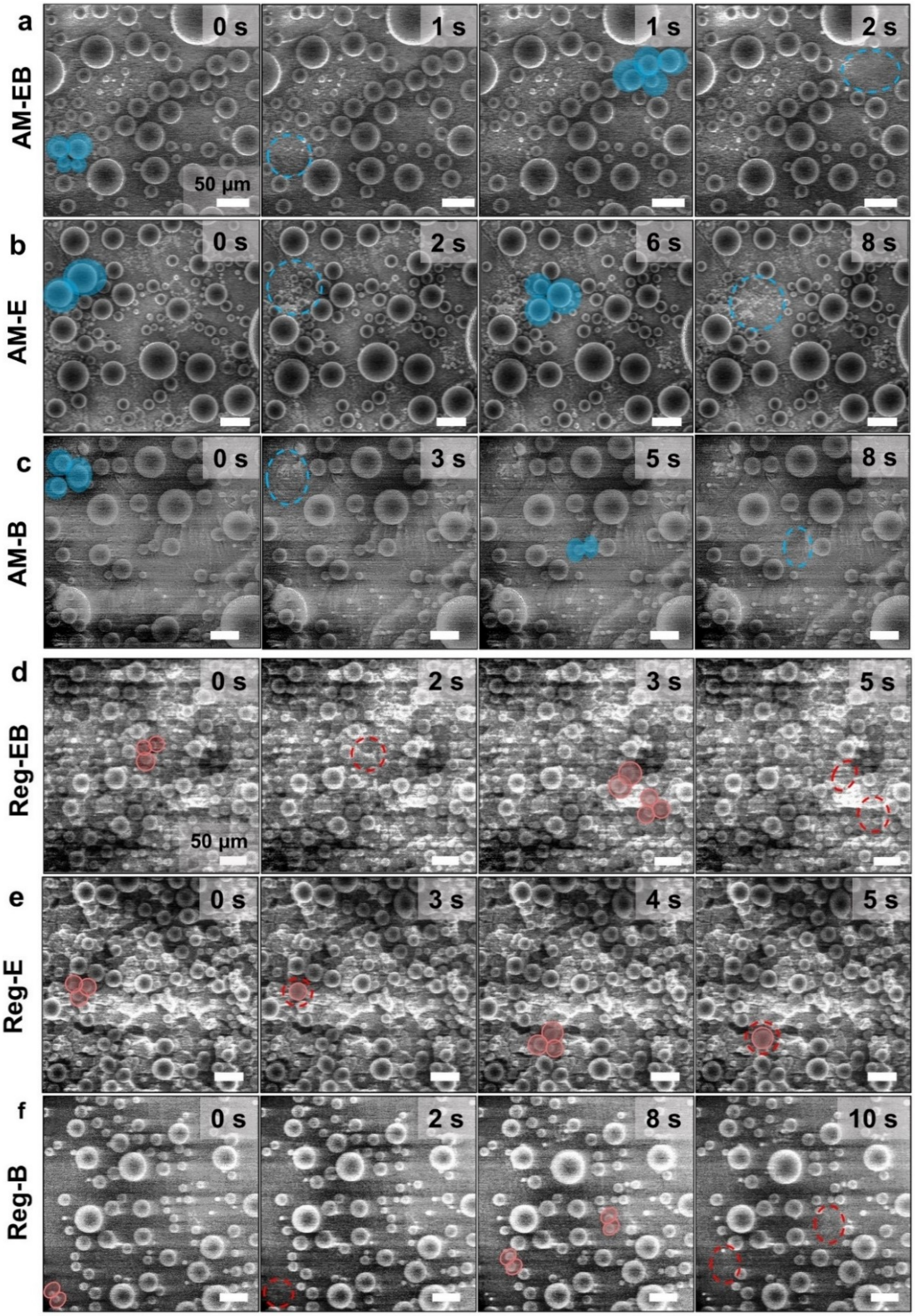

Figure S13. Time lapse of ESEM images for (a) AM-EB, (b) AM-E, (c) AM-B, (d) Reg-EB, (e) Reg-E and (f) Reg-B surfaces at S = 1.06. Jumping droplet condensation was observed on AM-EB, AM-E, AM-B, Reg-EB and Reg-B surfaces whereas no jumping event was observed for Reg-E surface.

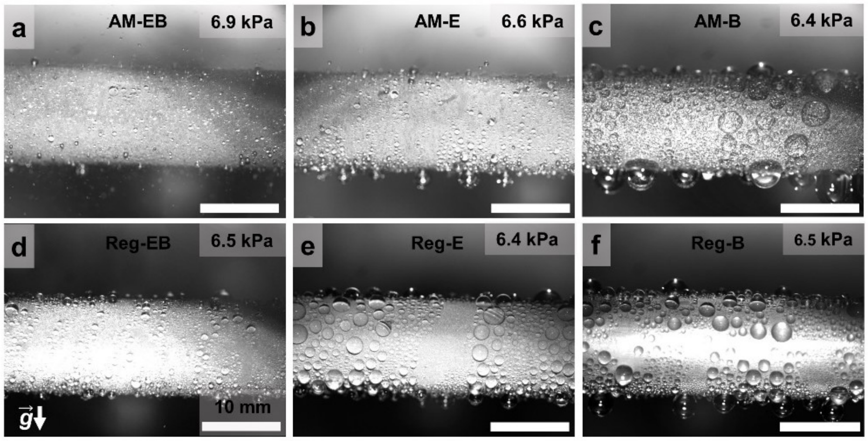

Figure S14. Exemplary optical high-speed images of pure steam condensation on various superhydrophobic surfaces at higher vapor pressures of 6.4-6.9 kPa. Only AM-EB and AM-B showed anti-flooding condensation while sustaining superior droplet jumping when compared to other tube samples. Scale bar in all images represents 10 mm.

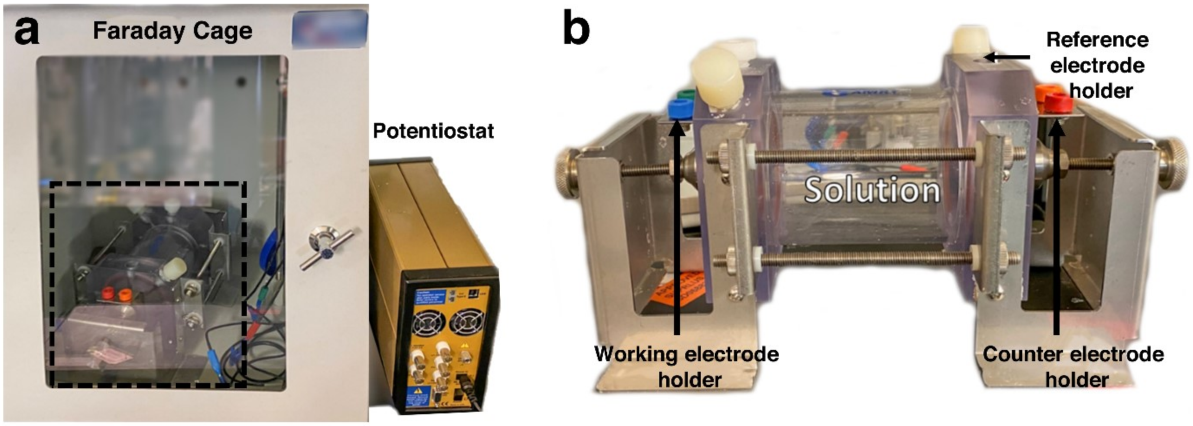

Figure S15. Photograph of the corrosion measurement setup. (a) Photograph of the potentiostat and the Faraday cage containing all the electrodes and electrical connections. (b) Photograph of the setup used for keeping a constant volume of the solution (~ 450 mL) and holding the reference electrode (Ag/AgCl Sat. KCl), counter electrode (Graphite), and working electrode (AM or Al test surfaces). 0.5 M NaCl was used as the solution.

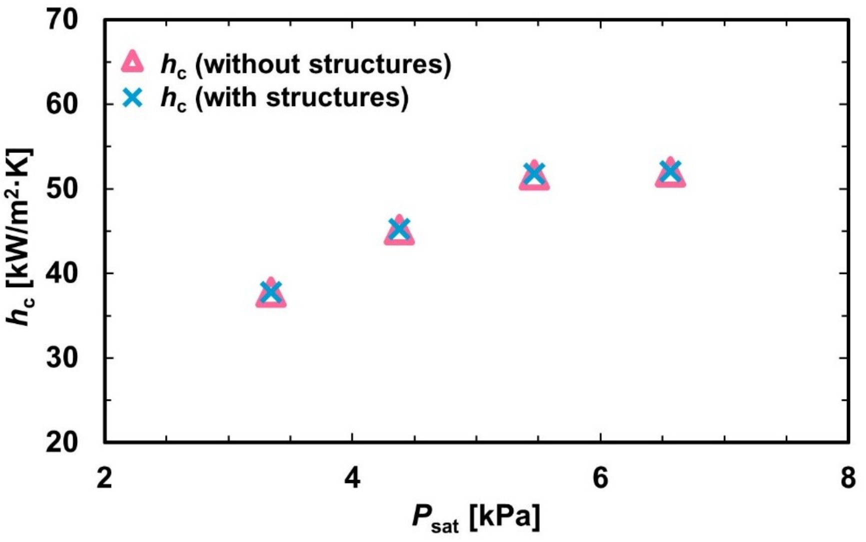

Figure S16. Effect of nanostructure thermal resistance on the h_c values of AM-E. The two heat transfer coefficient plots represent the measured heat transfer coefficients with and without taking the nanostructure thermal resistance into consideration.

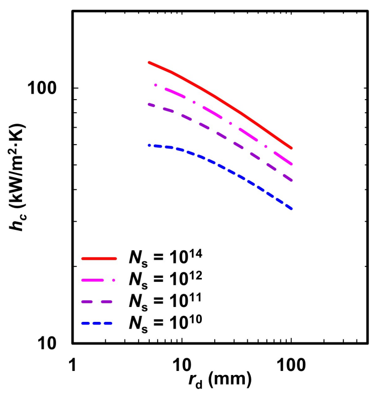

Figure S17. Effects of droplet departure radius (r_d) and nucleation density (N_s) on the theoretical condensation heat transfer coefficient (h_c).

Table S1. Details of test facility instrumentation and uncertainty of each measurement.

**Measurement**	**Instrument**	**Span**	**Uncertainty**
Coolant temperature	RTD	7-8°C	0.15°C ± 0.12%
Working fluid temperature	T-type thermocouple	20-100°C	±0.5°C
Coolant flow rate	Electromagnetic flow meter	11-30 L/min	±1%
Chamber pressure	Pressure transducer	0-17 kPa	±1%

Supplemental References

[1] Ho, J.Y., Leong, K.C., Wong, T.N., *Int. J. Heat Mass Transf.* **2020**, *150*, 119262.
[2] Ho, J.Y., Leong, K.C, Wong, T.N., *Int. J. Heat Mass Transf.* **2020**, *144*, 118599.
[3] Ho, J.Y., Leong, K.C., *Int. J. Heat Mass Transf.* **2020**, *150*, 119328.
[4] Ho, J.Y., Wang, X.W., Leong, K.C., Wong, T.N, *Int. J. Heat Mass Transf.* **2019**, *130*, 1004-1015.
[5] Wang, X.W., Ho, J.Y., Leong, K.C., Wong, T.N., *Int. J. Heat Mass Transf.*, **2018**, *126*, 949-962.
[6] Kempen, K., Thijis, L., van Humbeeck, J., Kruth, J.P., *Mater. Sci. Technol.* **2015**, *31*, 917-923.
[7] Kempen, K., Thijis, L., van Humbeeck, J., Kruth, J.P., *Phys. Procedia*, **2012**, *39*, 439-446.
[8] Rabbi, K.F., Boyina, K.S., Su, W., Sett, S., Thamban, A., Shahane, S., Wang, S., Miljkovic, N., *Int. J. Heat Mass Transf.* **2021**, *164*, 120563.
[9] Yan, Q., Song, B., Shi, Y., J., *Mater. Sci. Technol.* **2020**, *41*, 199-208.
[10] Shakil, S.I., Hadadzadeh, A., Amirkhiz, B.S., Pirgazi, H., Mohammadi, M., Haghshenas, M., *Results Mater.* **2021**, *10*, 100178.
[11] Sett, S., Sokalski, P., Boyina, K., Li, L., Rabbi, K.F., Auby, H., Foulkes, T., Mahvi, A., Barac, G., Bolton, L.W., Miljkovic, N., *Nano Lett.* **2019**, *19*, 5287-5296.
[12] Ho, J.Y., Rabbi, K.F., Sett, S., Wong, T.N., Miljkovic, N., *Int. J. Heat Mass Transf.,* **2021**, *172*, 121149.
[13] ASM International, Properties and selection: nonferrous alloys and special-purpose materials, Metals Handbook, 10th ed., vol. 2, The Materials Company, **1990**.
[14] Ho, J.Y., Wang, X.W., Leong, K.C., *Int. J. Heat Mass Transf.* **2018**, *126*, 652-666.
[15] Petukhov, B.S., Krasnoshchekov, E.A., Protopopov, V.S., Proc. 1961-1962 Heat Transf. Conf., University of Colorado, Boulder, USA, **1961**.
[16] Moffat, R.J., *J. Fluids Eng.* **1985**, *107*, 173-178.
[17] Nusselt, W., Die Oberflachenkondensation des Wasserdampfes. **1916**, *60*, 541-546 and 569-575.
[18] Colebrook, C.F., White, C.M., *Proc. R. Soc. A: Math. Phys. Eng. Sci.* **1937**, *906*, 367-381.
[19] Cha, H., Xu, C., Sotelo, J., Chun, J.M., Yokoyama, Y., Enright, R., Miljkovic, N., *Phys. Rev. Fluids* **2016**, *1*, 064102.
[20] Yan, X., Zhang, L., Sett, S., Feng, L., Zhao, C., Huang, Z., Vahabi, H., Kota, A.K., Chen, F., Miljkovic, N., *ACS Nano* **2019**, *13*, 1309-1323.
[21] Zhao, G., Zou, G., Wang, W., Geng, R., Yan, X., He, Z., Liu, L., Zhou, X., Lv, J., Wang, J., *Soft Matter* **2020**, *16*, 4462-4476.
[22] Rose, J.W., *Proc. Instn. Mech. Engrs*. **2002**, *216*, 115-128.
[23] Miljkovic, N., Enright, R., Wang, E.N., *J. Heat Transf.* **2013**, *135*, 111004.
[24] Kim, S., Kim, K.J., *J. Heat Transf.* **2011**, *133*, 081502.
[25] Rose, J.W., *Int. J. Heat Mass Transf.* **1981**, *24*, 191-194.
[26] Miljkovic, N., Enright, R., Nam, Y., Lopez, K., Dou, N., Sack, J., Wang, E.N., *Nano Lett.* **2013**, *13*, 179-187.
